# Wurtzite InP/ZnSe/ZnS Core/Shell Semiconductor Quantum Dots with Bright Near-IR Emission

**DOI:** 10.1021/jacs.6c05042

**Published:** 2026-06-15

**Authors:** Jiekai Dai, David Stone, Xiang Li, Adar Levi, Sergei Remennik, Uri Banin

**Affiliations:** † Institute of Chemistry, 26742 The Hebrew University of Jerusalem, Jerusalem 9190401, Israel; ‡ The Center of Nanoscience and Nanotechnology, 26742 The Hebrew University of Jerusalem, Jerusalem 9190401, Israel

## Abstract

Indium phosphide (InP) quantum dots (QDs) are already widely employed as heavy-metal-free materials for optoelectronic and bioimaging applications. However, common hot-injection methods produce *zinc-blende* phase InP QDs, and the synthesis of large-sized InP QDs with uniform size distribution and efficient near-infrared (NIR) emission has been limited. Here, starting from the cation-exchange synthesis of monodisperse *wurtzite* phase InP (w-InP) QDs with tunable size, we report the epitaxial growth of ZnSe/ZnS shells to significantly enhance photoluminescence (PL) efficiency and photochemical stability. The resulting w-InP/ZnSe/ZnS core/shell/shell (CSS) QDs exhibit narrow size-tunable bright NIR emission (∼740–820 nm). For example, for cores in the midrange (d = 8.7 ± 0.7 nm; peak emission wavelength at ∼780 nm), the PL quantum yield (QY) reaches 78%, with a narrow full-width at half-maximum (fwhm) of ∼33 nm (∼69 meV). Photostability studies reveal that the optical properties of both the w-InP core and the CSS QDs remain stable in ambient conditions under dark storage. Under illumination, the w-InP cores show increased PLQY due to light-induced surface oxidation, while in the presence of oxygen, CSS QDs experience a decline in PL performance due to photo-oxidation of the ZnSe shell. This degradation is lessened upon exposure to shorter wavelength light, suggesting the involvement of outer shell states in this process. These high-performance, cadmium-free NIR-emitting QDs thus hold strong potential for applications in advanced optoelectronics and bioimaging technologies.

## Graphical abstract

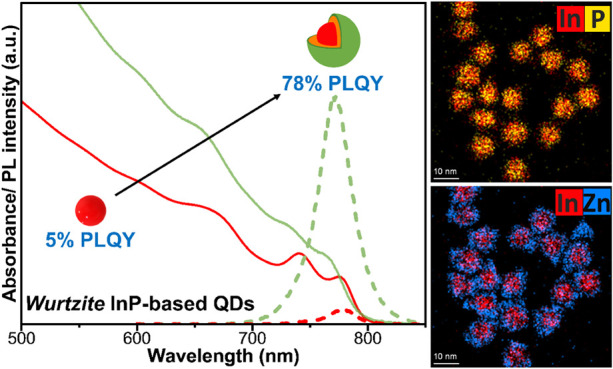

## Introduction

Visible to near-infrared (NIR) light-emitting strongly semiconductor colloidal quantum dots (QDs) are of direct applicative significance for display technologies,^1,2^ bioimaging,^3,4^ and optoelectronics.^5,6^ In the past few decades, Cd-chalcogenide QDs have been extensively studied due to their well-controlled synthesis, tunable emission wavelengths across the entire visible spectrum^7^ and high photoluminescence (PL) quantum yield (QY).^8–10^ However, their widespread commercialization has been restricted by environmental and health concerns, particularly due to heavy-metal toxicity and limitations imposed by the RoHS (Restriction of Hazardous Substances) regulation.^11–13^ In this context, III–V semiconductor QDs, including indium arsenide (InAs) and indium phosphide (InP), have emerged as favorable, less toxic alternatives,^14–17^ while achieving bright emission beyond **700 nm** via InP QDs with narrow line width remains challenging due to the strong covalent bonding in III–V materials^18–20^ and the high reactivity of commonly employed phosphorus precursors.^21^ These factors complicate size control and typically lead to size polydispersity, impeding the development of InP QDs with narrow and efficient NIR emission.^22^ Moreover, the synthesis of anisotropic phases of InP QDs is also restricted, as the hot-injection method yields only the thermodynamically favored isotropic *zinc-blende* phase InP QDs.

An alternative route that can yield anisotropic *wurtzite*-phase InP (w-InP) QDs utilizes Cu-to-In cation exchange from hexagonal copper phosphide (Cu_3–*x*_P) QDs,^23,24^ and early work demonstrated this route on large hexagonal plate structures that did not manifest bandgap emission. More recently, high-quality luminescent anisotropic w-InP core QDs were synthesized with outstanding control over size and emission characteristics.^25–27^ This was enabled by synthesizing the ‘mother’ Cu_3–*x*_P QDs via a two-step process, forming at first well-controlled monodisperse Cu nanoparticles, followed by their phosphidation to Cu_3‑x_P QDs. Following the cation exchange of Cu to In, a postsynthetic surface reaction using nitrosyl tetrafluoroborate (NOBF_4_) was further introduced, which enabled the extraction of remaining copper impurities and the removal of surface oxides. The resulting w-InP QDs manifested sharp absorption features alongside bandgap PL. This approach so far enabled the preparation of w-InP QDs with diameters ranging from 5 to 12 nm, corresponding to emission wavelengths spanning 650–820 nm—an NIR range that remains difficult to access through conventional hot-injection synthetic routes.^25,26,28–30^ However, prolonged etching compromises size monodispersity, and while a PLQY of up to 40% could be achieved by the surface reaction, it resulted in the broadening of the size distribution and PL width due to the prolonged etching times.^26,31^ Moreover, this process is also inherently accompanied by a reduction in QD size and a related blue shift of the PL, limiting the ability to achieve a desired emission wavelength. Herein, we address this limitation by introducing the growth of w-InP/ZnSe/ZnS core/shell/shell (CSS) QDs, with high PLQY at controlled central emission wavelengths and narrow PL fwhm.

Core/shell architectures are widely applied, where the core QD is passivated by shell materials with a wider bandgap. Proper shell growth not only spatially confines charge carriers within the core but also passivates surface trap states, thus enhancing both high PLQY and long-term photochemical stability while maintaining narrow PL features.^32^ To this end, the selection of shell materials should generally favor type-I band alignment to confine the electron and hole to the shell-protected core region. Furthermore, lattice compatibility is typically sought to minimize interfacial strain. In particular, ZnSe, ZnSeS, and ZnS have been applied as effective shell materials for the common *zinc-blende* InP- and InAs- based QD systems.^16,33–39^ Such a strategy was successful in achieving green- and red-emitting InP/ZnSe/ZnS double-shell structures with *zinc-blende* InP cores, near-unity PLQY, and enhanced thermal and photochemical stability, highlighting the effectiveness of shell-engineering strategies for optical improvement.^13,33,34^ Recent studies show that giant InP/ZnSe/ZnS and InP/ZnSe/ZnSeS/ZnS QDs (13.4–20.8 nm) can yield PLQY values exceeding 90% (emitting at 606–630 nm) when shell thickness and composition are precisely controlled; slower shell growth and carefully tailored ZnSe/ZnSeS/ZnS layer sequences enable highly efficient 20.8 nm QDs, while sulfur-rich shells tend to form shield-like morphologies that may alleviate strain during shell growth.^36^ However, for type-I core/shell heterostructures, despite the enhanced optical performance and stability, thick shells provide only limited red-shifts in emission while reducing size uniformity and increasing strain effects at interfaces. Furthermore, for NIR-emitting *zinc-blende* InP QDs, InP/ZnS synthesized from 16 nm cores have been reported to emit at 807 nm; however, they exhibit a low PLQY of ∼12% and a broad emission peak (fwhm = 111 nm), indicating minimal photophysical improvement and compromised size homogeneity—a persistent bottleneck in conventional InP QD synthetic routes.^40^

In contrast, high-quality w-InP-based core/shell QDs have yet to be developed. A sole example of a prior core/shell system on w-InP focused on the 600–700 nm emission regime, and by growing a ZnSeS alloy shell^41^ only a limited PLQY of ∼4% was achieved. These results indicate the need to develop suitable CSS QD structures with w-InP cores that exhibit strong PLQY and narrow fwhm with emission extending to the NIR region. In this work, we report the synthesis of highly NIR-emissive *wurtzite* InP/ZnSe/ZnS core/shell QDs. Focusing on QDs with NIR emission between 740–820 nm, we were able to achieve a PLQY of ∼78% for QDs with an emission peak at 770 nm and a remarkably narrow fwhm of ∼33 nm (∼69 meV). Systematic investigation revealed a strong dependence of the final PLQY on the initial optical quality of the w-InP cores, underlining the significance of removing copper impurities and surface oxides prior to shell growth, as well as the critical influence of core/shell interface quality. Optimal synthetic conditions were also identified, focusing on the growth temperature and the colloidal chemical environment. Notably, elevated temperatures during ZnSe shell growth and an intermediate purification step prior to ZnS shell deposition significantly improved shell morphology and PL performance. Importantly, the core/shell architecture preserves the **wurtzite** crystal structure of the InP core, indicating minimal structural transformation during shelling. Photostability studies, accompanied by surface characterization via X-ray photoelectron spectroscopy (XPS), confirmed their improved resistance to oxygen exposure and photo-oxidation, which was found to depend on the incident light wavelength. For illumination at wavelengths exceeding the direct absorption into the ZnSe shell states, better stability was observed. Overall, this work provides a comprehensive synthetic strategy for producing highly emissive w-InP/ZnSe/ZnS QDs, offering promising potential for NIR optoelectronic devices and bioimaging applications.

## Results and Discussion

In order to overcome the long-standing limitations in synthesizing large, monodisperse, and efficiently NIR-emissive w-InP QDs, we designed a synthetic strategy that integrates the cation-exchange approach with controlled heteroepitaxial shell growth (reaction scheme illustrated in Figure 1a). Briefly, the process begins with the synthesis of templating hexagonal Cu_3–*x*_P QDs, followed by a cation-exchange reaction to form w-InP QDs. These QDs are then subjected to a surface reaction using a NOBF_4_-DMF solution to yield emissive w-InP QDs with well-resolved absorption features and narrow NIR PL. Finally, gradient shell passivation was carried out to produce highly fluorescent w-InP/ZnSe/ZnS QDs. Detailed procedures are provided in the Materials and Methods section.

**Figure 1:**
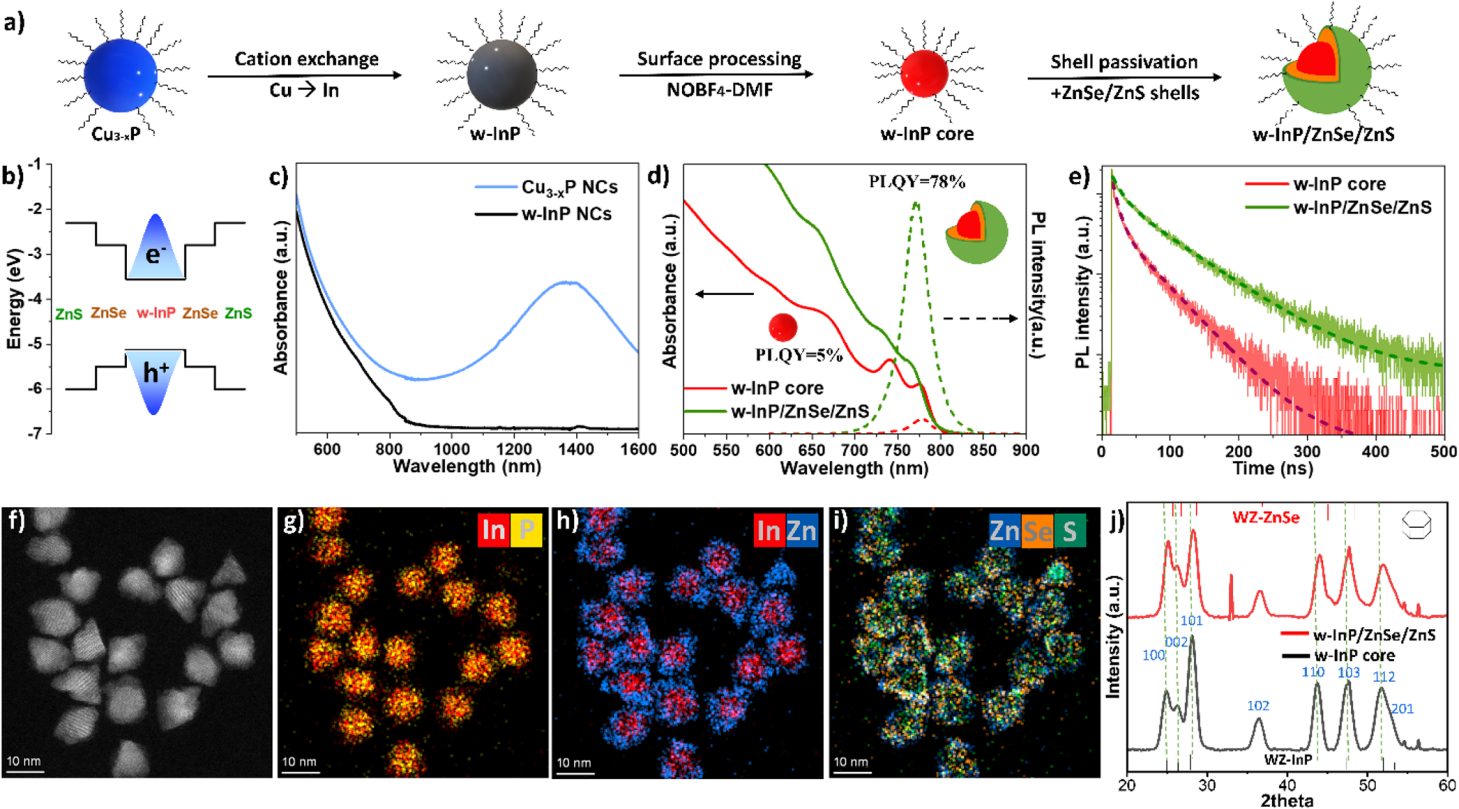
Preparation and characterization of w-InP/ZnSe/ZnS QDs: (a) Schematic illustration of the synthetic route: Cu_3–*x*_P QDs are transformed into w-InP QDs via cation exchange, treated with NOBF_4_, and passivated with epitaxial ZnSe/ZnS shells. (b) Potential energy scheme for w-InP/ZnSe/ZnS core/shell/shell QDs. (c) Absorption of the mother Cu_3–*x*_P QDs (blue) and the w-InP QDs after cation exchange (black), showing the disappearance of the plasmon peak centered at 1400 nm and the appearance of the InP absorption onset at ∼830 nm. (d) Absorption (solid lines) and PL (dashed lines) of the w-InP core QDs after the surface reaction, manifesting sharp absorption features (red) and the CSS QDs (green), reaching the high PLQY of 78% (excitation wavelength of 500 nm). (e) Time-resolved photoluminescence (TRPL) decay traces comparing cores (red) and core/shells (green) (biexponential fitting shown as dashed lines). (f–i) HAADF-STEM image (f) of w-InP/ZnSe/ZnS and corresponding mapping of elements of multiple QDs based on EDX scan: (g) In, P; (h) In, Zn; (i) Zn, Se, S. (j) X-ray diffraction (XRD) patterns of w-InP and w-InP/ZnSe/ZnS.

To achieve high PLQY, ZnSe and ZnS were chosen as shell materials and epitaxially grown on the w-InP cores to form a type-I band alignment (Figure 1b), while gradually transitioning the lattice spacing from InP to ZnSe to ZnS. Starting with hexagonal Cu_3–*x*_P QDs, cation exchange produced w-InP QDs, as evidenced by the disappearance of the broad plasmon peak at ∼1400 nm, which is due to copper vacancies leading to free holes, and the appearance of an absorption onset around 830 nm. This confirms the formation of w-InP via cation exchange of Cu­(I) by In­(III) (Figure 1c). TEM images and corresponding size histograms of Cu_3‑x_P (11.5 ± 1.0 nm) and w-InP QDs (11.2 ± 0.9 nm) are presented in Figure S1, demonstrating the size preservation and excellent size uniformity after the cation exchange reaction. However, the as-prepared w-InP QDs exhibit a featureless absorption spectrum and no detectable PL, primarily due to residual copper impurities and surface defects.

Surface treatment with NOBF_4_-DMF solution at room temperature for 1 h yielded luminescent w-InP QDs with distinct excitonic features in the absorption spectrum (Figure 1d), confirming the removal of remaining copper impurities and surface oxides. The resulting QDs (average diameter of ∼8.7 nm) emit in the NIR region, with an emission peak centered at ∼780 nm, a PLQY of ∼5%, and a narrow fwhm of ∼27 nm (∼55 meV). Extending the etching time further enhanced the PLQY, reaching up to ∼40%,^26^ but this was accompanied by a widening of the size distribution, leading to smeared absorption features and a broadened emission peak. Therefore, to maintain size homogeneity, we utilized an optimal moderate etching time (1 h at RT, see below) followed by heteroepitaxial shell growth on the w-InP QDs. Following shell passivation with ZnSe and ZnS, highly luminescent w-InP/ZnSe/ZnS CSS QDs were obtained, emitting at ∼770 nm with ∼78% PLQY and ∼33 nm (∼69 meV) fwhm (Figure 1d). The negligible Stokes shifts for both the core and CSS QDs are consistent with the narrow size distribution alongside a small excitonic fine-structure splitting expected in such large core QDs.

To assess the influence of shell passivation on carrier recombination dynamics, time-resolved photoluminescence (TRPL) measurements were performed. As shown in Figure 1e and Table S1, the average PL lifetime (τ_avg_) increases from 36 ns (core-only) to 66 ns (CSS). While τ_avg_ nearly doubles, this increase cannot fully account for the ∼15-fold enhancement in PLQY using a homogeneous kinetic framework. This indicates that the recombination is governed by a heterogeneous kinetics model, in which the effective surface passivation by shell growth also greatly increases the fraction of emissive QDs. The PL decay curves are well-fitted by a biexponential model, also revealing inhomogeneous excitonic subpopulations, where the short-lived component (τ_1_) is assigned to trap-mediated nonradiative recombination, and the long-lived component (τ_2_) represents radiatively efficient band gap emission. The substantial increases in τ_1_ and the average lifetime (τ_avg_) upon ZnSe/ZnS shell growth indicate a marked suppression of nonradiative decay pathways and an increased contribution from the radiative subpopulation. These findings, consistent with the enhanced PLQY, confirm that the dual-shell structure effectively passivates surface defects and promotes radiative exciton recombination across the ensemble.

High-resolution high-angle annular dark-field scanning transmission electron microscopy (HAADF-STEM) imaging and elemental mapping via energy-dispersive X-ray (EDX) analysis (Figures 1f–i and Figure S2 for a cross-section analysis) confirm the formation of a well-defined w-InP/ZnSe/ZnS core/shell/shell architecture. Indium and phosphorus atoms are localized in the core region, while zinc, selenium, and sulfur are distributed in the surrounding shell, consistent with the intended compositional gradient. Notably, while the w-InP cores exhibit a rounded prismatic-hexagonal shape, the core/shell particles display a less regular shape. This evolution is attributed to facet-dependent shell growth. For wurtzite InP QDs, different crystallographic facets (basal and prismatic planes) possess distinct surface energies and reactivities, resulting in anisotropic shell deposition. The use of a highly reactive Zn­(OA)_2_ precursor further promotes rapid Zn incorporation at the early stages of growth, which is likely to occur preferentially on indium-rich, defect-free facets, thereby enhancing facet-selective deposition. In addition, lattice mismatch and the associated interfacial strain between the core and shell materials can further influence facet-dependent growth rates. As shell growth progresses, these combined effects lead to increasing anisotropy and deviation from the initial quasi-spherical morphology. High-resolution STEM analysis of single nanoparticles presents both (100) (prismatic) and (002) (basal) facets, supporting this facet-dependent kinetic growth. As shown in Figure S3, depending on the viewing direction, projections along the *c*-axis yield a more symmetric, quasi-spherical appearance, whereas projections along prismatic directions reveal a more anisotropic morphology. XRD patterns of both w-InP cores and w-InP/ZnSe/ZnS CSS QDs (Figure 1j) reveal the preservation of the wurtzite crystal structure after shell growth. Notably, the diffraction peaks of w-InP/ZnSe/ZnS CSS QDs shift toward higher angles, approaching those characteristic of w-ZnSe, consistent with coherent epitaxial shell growth. The relative peak intensities, particularly for the first three diffraction angles, suggest a preferential orientation of the QDs on the substrate. Specifically, the results present a higher contribution from pyramidal (101) and prismatic (100) facets compared to the hexagonal basal (002) facet. Regarding the peak line widths, no significant change in the fwhm is observed after shell growth. This is likely due to the relatively large size of the InP cores and the limited thickness of the shell, resulting in only a minor contribution of the shell to peak fwhm alteration. In addition, the minor sharp peaks at 30°–35° and beyond 55° are related to background substrate signals.

To illustrate the necessity of NOBF_4_-based surface processing prior to shell growth, Figure S4 presents the optical and structural evolution of cation-exchanged QDs during the shelling process. The initial w-InP sample, which had **not** undergone any postsynthetic treatment to remove residual copper impurities or oxides, exhibits broad and poorly resolved excitonic absorption features and no PL, indicating a less effectively passivated surface and compromised electronic structure. Even after ZnSe deposition, the PL improvement is negligible, with the final PLQY reaching only ∼0.3%. TEM analysis corroborates these findings: the particle size increases only from ∼8.4 to ∼9.6 nm, corresponding to an ultrathin (<2 MLs) ZnSe shell, far thinner than the ∼5 MLs expected from the added precursor amount. The limited size growth and minimal PL enhancement together demonstrate that residual impurities and surface oxides inhibit uniform shell growth, leading to poor shell integrity and a defective core/shell interface. These observations underscore the need for an effective surface treatment step prior to shelling.

To address this issue, the application of the surface treatment via reaction with NOBF_4_ prior to shell growth was studied by examining the relationship between the initial PLQY after etching and the final PLQY after shell passivation. This correlation is found to be a key determinant of effective shelling. The results demonstrate that a higher final PLQY is generally associated with a higher initial PLQY (Figure 2a). The higher initial PLQY reflects the removal of copper impurities and better surface quality of the w-InP cores, with reduced surface oxides and fewer surface traps. These features facilitate efficient epitaxial shell growth while minimizing interfacial core/shell traps, thereby enhancing the final PLQY. However, a maximal limit to this correlation is observed: at extreme levels of surface etching, even with an initial PLQY as high as ∼20%, the final PLQY does not show further improvement. This may be attributed to excessive surface fluorination, which may block reactive sites on the core surface and hinder the adsorption of shell precursors. In addition, it was reported that, regarding strong fluoride treatment, the formation of Zn–F is more accessible than ZnSe considering their Gibbs free energy,^36^ leading to insufficient inner shell passivation. Moreover, prolonged reaction with NOBF_4_ etches the cores and broadens the size distribution and hence the fwhm. The conclusion from this study is that w-InP cores with an initial PLQY of 5–10% are best suited for optimal subsequent shell growth to yield high final PLQY and narrow PL fwhm.

**Figure 2:**
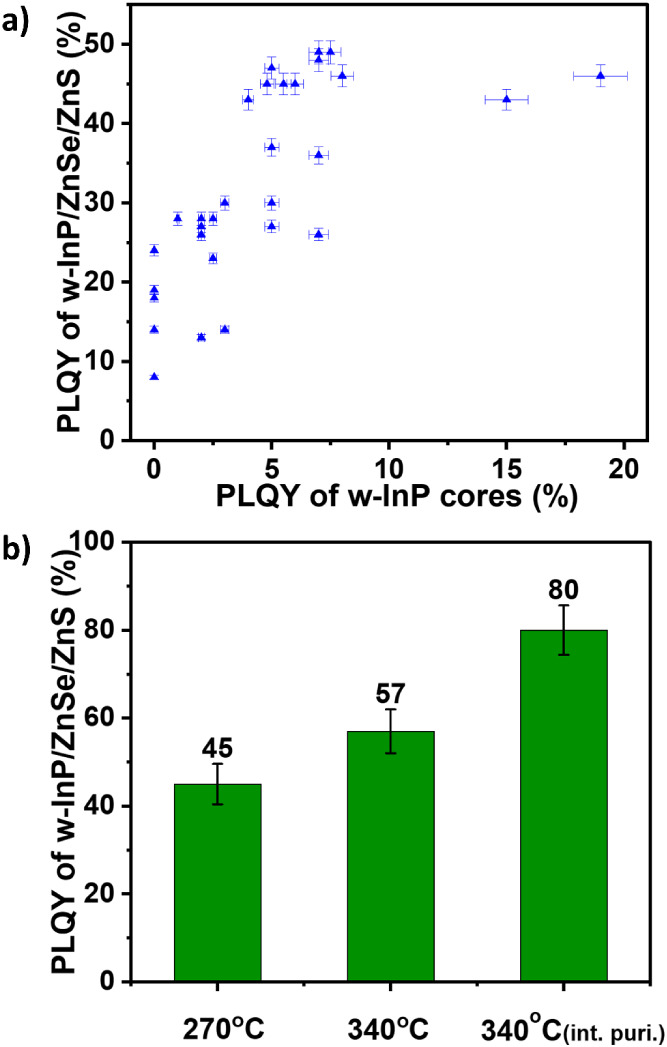
Study of synthetic conditions: (a) The relation of w-InP core initial PLQY versus w-InP/ZnSe/ZnS CSS QDs final PLQY. (b) Effect of shell growth conditions, including reaction temperature and intermediate purification (int. puri.) steps, to obtain improved PLQY and shell morphology (HR-STEM image of w-InP/ZnSe/ZnS without intermediate purification is in SI ).

Focusing our efforts on the shell growth conditions onto w-InP cores with an initial PLQY of 5–10% (Figure 2b), we further studied the effects of shell growth conditions. First, elevating the ZnSe shell growth temperature from 270 °C to 340 °C enhances the PLQY to ∼57%. Raising synthetic temperatures can promote greater atomic mobility,^33^ which facilitates improved annealing and crystallinity, and hence more effective surface passivation. Moreover, temperature plays a critical role in regulating the dynamic equilibrium between ligand adsorption and desorption, which is essential for achieving uniform growth and precise shape control.^34,42,43^

Second, we found that introducing an intermediate purification step after the ZnSe shell growth and prior to ZnS deposition leads to a significant improvement in both optical and structural properties, elevating the PLQY to ∼80% and yielding a more controlled shell morphology (Figure S5). Intermediate purification enables more uniform attachment of shell materials to the w-InP cores, rather than fragmented deposition, thereby improving PL efficiency. This enhancement is primarily attributed to the removal of unreacted precursors, excessive ligands, and byproducts that could disrupt the surface chemistry and impede epitaxial shell material growth.^13,43^ It enables more controlled shell growth while minimizing interfacial defects, which is crucial for achieving highly luminescent and uniformly passivated w-InP-based QDs.

Regarding the selection of shell precursors, particularly the zinc precursors, the reactivity is primarily tuned by the oleic acid-to-zinc cation ratio, which modulates the coordination environment and the effective availability of Zn^2+^ ions in the reaction system. A higher oleic acid content leads to stronger ligand coordination to Zn^2+^, increasing its solubility but reducing its effective chemical reactivity because of the decreased availability of reactive species.^44^ This lower reactivity is advantageous for ZnS outer shell growth, where controlled and thermodynamically driven deposition is required to achieve a uniform and epitaxial coating. In contrast, a higher-reactivity zinc precursor, with reduced ligand coordination, provides a higher concentration of reactive Zn species and supports faster kinetically driven growth. This condition is particularly beneficial for ZnSe shell formation, where rapid nucleation and growth facilitate efficient surface passivation, resulting in a pronounced increase in the PLQY at early growth stages.

Figures 3a,b shows the evolution of absorption and PL spectra, along with the corresponding PLQY, PL peak wavelengths, and fwhm during ZnSe and ZnS shell growth on w-InP cores (8.7 ± 0.7 nm). The w-InP core QDs’ absorption exhibits well-resolved transitions, indicating high size uniformity, as further confirmed by the narrow emission line width (λ_em_ ∼ 780 nm, fwhm ∼ 27 nm) and the TEM images in Figure 3c. Prior to shell growth, an in situ ZnF_2_ surface treatment is applied, which generates HF in situ to remove surface oxides and improve epitaxial growth,^15,45^ resulting in a slight blue shift in the PL peak from ∼780 to ∼770 nm, a PLQY enhancement from ∼5% to ∼19%, and a slight change in particle size (8.9 ± 0.6 nm, Figure S6). Upon deposition of 2.5 MLs of ZnSe, the PL peak exhibits a slight red shift from 770 to 773 nm, and the PLQY increases to ∼43%. Further increasing the ZnSe shell thickness to 3.5 MLs (Figure 3d) enhances the PLQY to ∼62%. The subsequent addition of 1 ML of a ZnS outer shell (Figure 3e) leads to a final PLQY of ∼78% (after purification).

**Figure 3:**
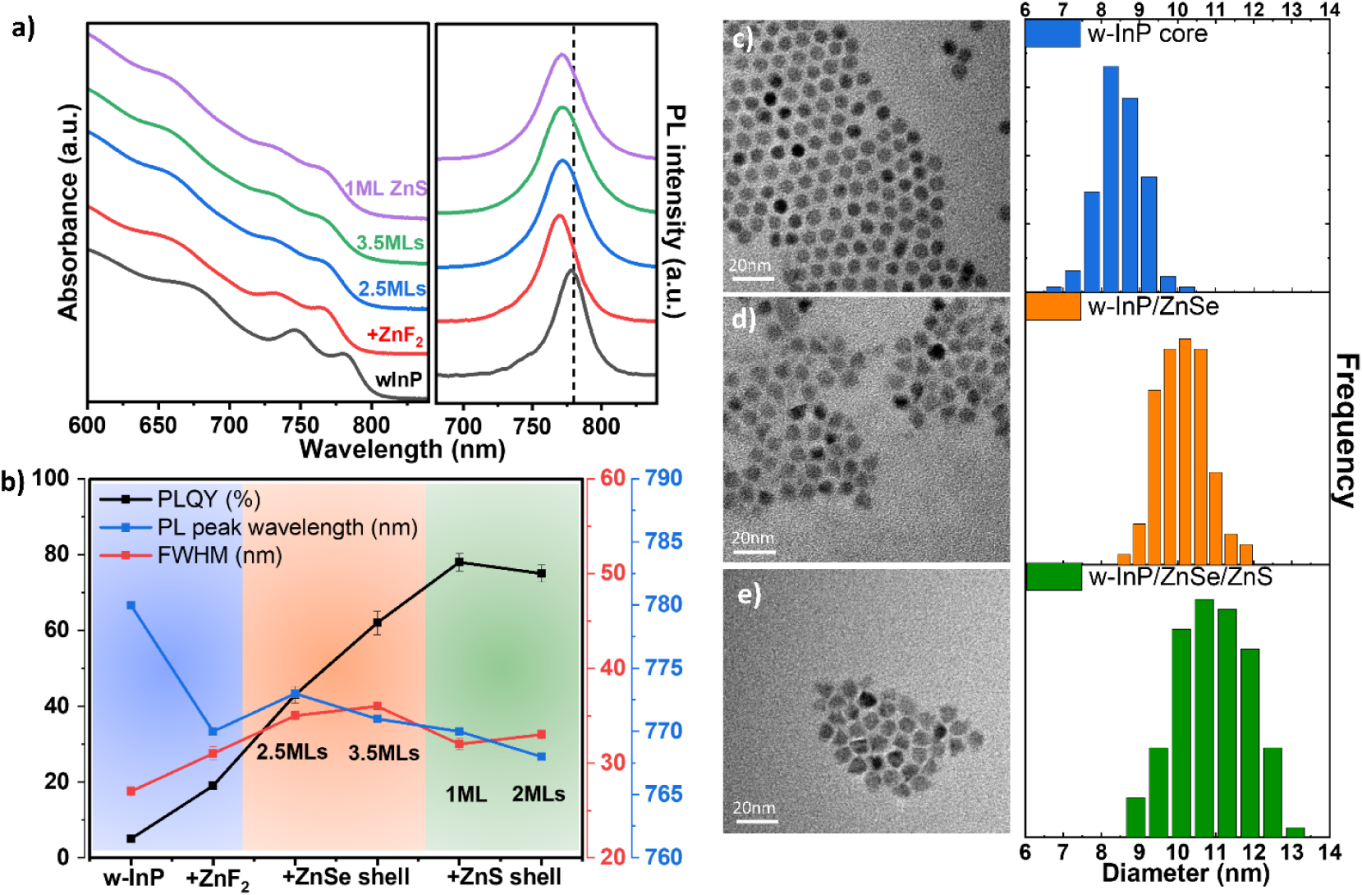
Evolution of optical and structural properties during shell growth on w-InP cores: (a) UV–vis–NIR absorption and PL spectra (black line: w-InP core after NOBF_4_ surface processing; red line: ZnF_2_ in situ treatment before shell growth; blue line: w-InP/(2.5 MLs)­ZnSe; green line: w-InP/(3.5 MLs)­ZnSe; purple line: w-InP/(3.5 MLs)­ZnSe/(1 ML)­ZnS; black dashed line: peak emission wavelength (∼780 nm) of w-InP core) (b) Evolution of PLQY (black squares, left axis), PL peak wavelength, and fwhm (blue and red squares, respectively, right axis) during the w-InP/ZnSe/ZnS CSS QDs. (c-e) TEM images and size distribution histograms of w-InP core, w-InP/ZnSe, and w-InP/ZnSe/ZnS.

To further optimize the CSS system, the effect of shell thickness on PLQY was studied (Figure S7). As shown in Figures S7a,b, increasing the ZnSe shell thickness to 7.3 MLs causes an observable red shift of the PL peak, accompanied by a notable PLQY decrease from ∼64% to ∼40% and a broadening of the emission spectrum. This PL deterioration is likely attributed to an Ostwald ripening-dominated growth regime under prolonged high-temperature conditions, as well as strain accumulation. These factors can lead to poor size distribution, trap formation, and electron delocalization, resulting in decreased QY and PL broadening.^46,47^

Based on the optimized w-InP/ZnSe (3.5 MLs) core/shell structure, the impact of the ZnS outer shell thickness was also investigated (Figures S7c,d). A thicker ZnS shell (4 MLs) results in a slight blue shift of the emission peak and a decrease in PLQY from ∼78% to ∼60%. The blue shift can be attributed to alloying effects, where the thicker shell layers can form a ZnSeS composition, leading to enhanced quantum confinement. Additionally, as the shell becomes thicker, the accumulated strain may be influential, potentially compromising the uniformity of the shell structure.^48^ This can induce the formation of trap states, which act as nonradiative recombination sites and reduce PLQY.

To evaluate the practical applicability of the NOBF_4_-treated w-InP cores and the resulting w-InP/ZnSe/ZnS CSS QDs, their intrinsic photostability was systematically evaluated under four conditions: in the dark and under continuous illumination, each conducted in both inert and ambient environments for 6 h. Changes in fluorescence intensity, as indicated by PLQY over time, along with corresponding PL lifetime measurements before and after light and air exposure, are presented in Figure 4. As shown in Figure 4a, the w-InP cores exhibit excellent stability in the dark under both inert and ambient conditions, with no significant change in PL intensity. Interestingly, under continuous illumination, the PLQY increased in both environments. In particular, in ambient air, the PLQY rose significantly from ∼5% to ∼19% after 6 h. The PL lifetime (Figure 4b), fitted with a biexponential model, shows that while the lifetimes τ_1_ and τ_2_ remained nearly unchanged (from 11.0 to 13.0 ns and 55.4 to 57.5 ns, respectively), the relative contribution of the longer lifetime pre-exponential factor (A_2_/A_1_) increased notably from 0.57 to 0.70. This suggests more efficient radiative recombination following QD surface photo-oxidation, consistent with the observed PLQY enhancement (Table S2).

**Figure 4:**
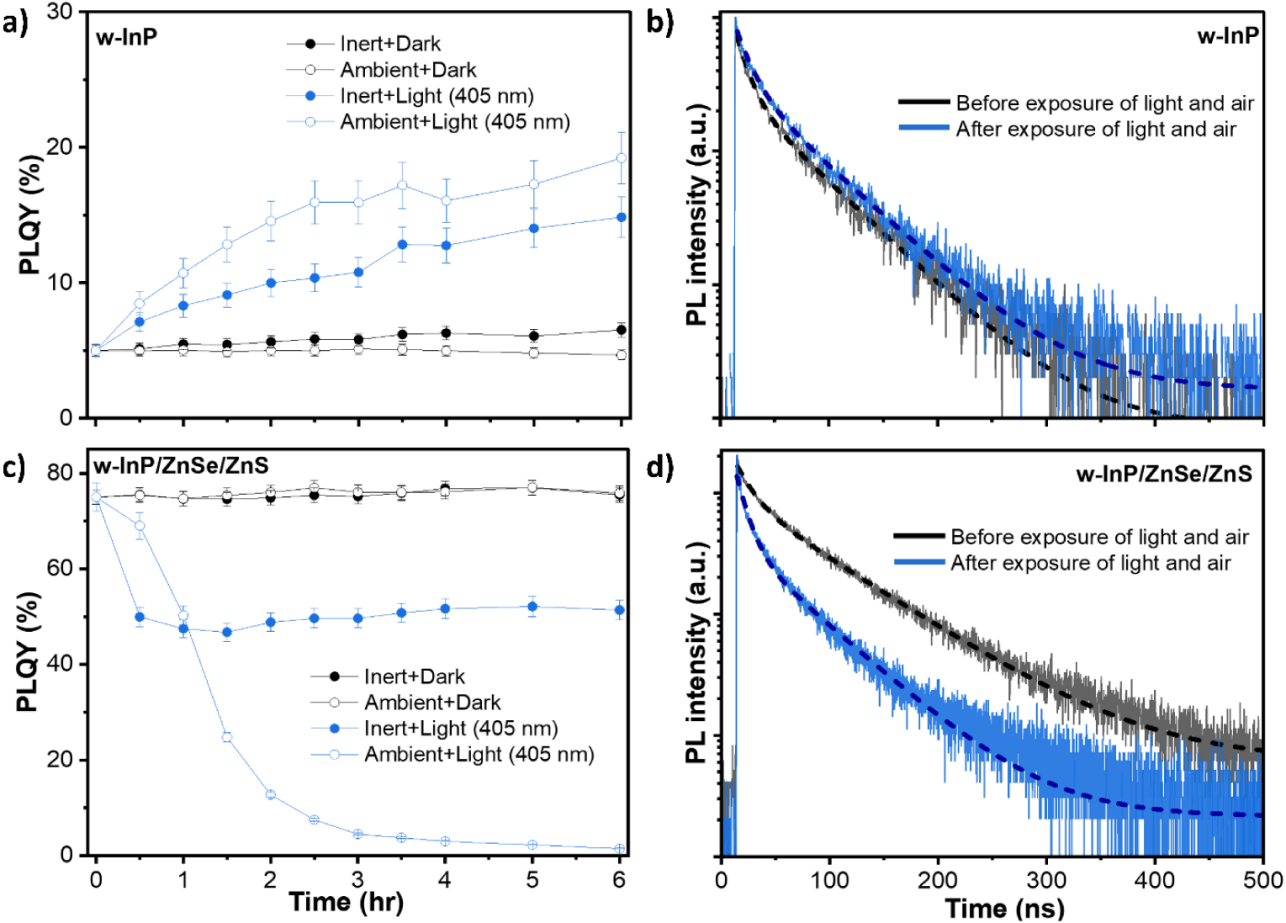
Photostability study of w-InP core and w-InP/ZnSe/ZnS QDs: (a, c) PLQY versus time under four specific conditions (inert + dark; ambient + dark; inert + light (405 nm); ambient + light (405 nm)) for w-InP core QDs (bandgap emission peak of ∼780 nm) and for w-InP/ZnSe/ZnS QDs, respectively. (b, d) PL lifetime measurements before and after exposure to ambient atmosphere and illumination (405 nm LED) for 6 h (biexponential fitting shown as dashed lines) of w-InP core QDs and w-InP/ZnSe/ZnS QDs, respectively.

For the w-InP/ZnSe/ZnS QDs, Figure 4c also shows high stability in the dark under both inert and ambient environments. However, under continuous illumination, a drop in PL intensity was observed. In the inert environment, the PLQY decreased from 78% to ∼50% within the first 30 min and then stabilized. Moreover, under ambient conditions, the PL intensity decreased rapidly and was nearly quenched after 6 h. This degradation is demonstrated by the PL decay curves (Figure 4d), which show a significant reduction in the average lifetime (τ_avg_) from 66 to 40 ns and the shorter lifetime (τ_1_) from 14.5 to 9.7 ns, consistent with trap state formation and pronounced nonradiative recombination (Table S3).

In addition, stability investigations were conducted on core/shell samples with a thicker outer ZnS shell (∼3 MLs instead of ∼1 ML), as shown in Figure S8. These results indicate that increasing the ZnS shell thickness leads to a reduction in final PLQY (from 78% to 55%) but improves resistance to photobleaching under photo-oxidative conditions, demonstrating that protective ZnS layers mitigate the light-induced interfacial oxidation effect.

To further investigate the surface chemical states and gain insight into the mechanisms underlying atmospheric and photoinduced changes in PL intensity, an XPS study was conducted. Starting with w-InP cores, the F 1s spectra display two distinct binding energy peaks: 684 eV for fluorine coordinated with metal ions (F–In; denoted F 1s (I)) and 687 eV for fluorine in organic environments (F–C; denoted F 1s­(II)) (Figure 5a). After a 6-h exposure to light and air, the F 1s­(II) peak exhibits a noticeable shift toward higher binding energy, accompanied by an increase in the relative intensity of the F 1s­(I) peak from 39% to 61%. This variation suggests a surface chemical reconfiguration. Under photooxidative conditions, residual BF_4_^–^ species are destabilized, enabling fluorine to form stronger, more stable bonds with In atoms on the w-InP surface sites. The shift in F 1s­(II) reflects reduced local electron density, consistent with the formation of more ionic In–F species.^28,47,49,50^ In addition, an increase in In–O signals (Figure 5b) from 5% to 19% indicates the formation of a thin, protective In_2_O_3_ layer on the core w-InP surface. Together, the fluoride and oxide bonding to the core surface thus improve surface passivation, suppress nonradiative recombination, and thereby contribute to the observed increase in PLQY of w-InP cores.

**Figure 5:**
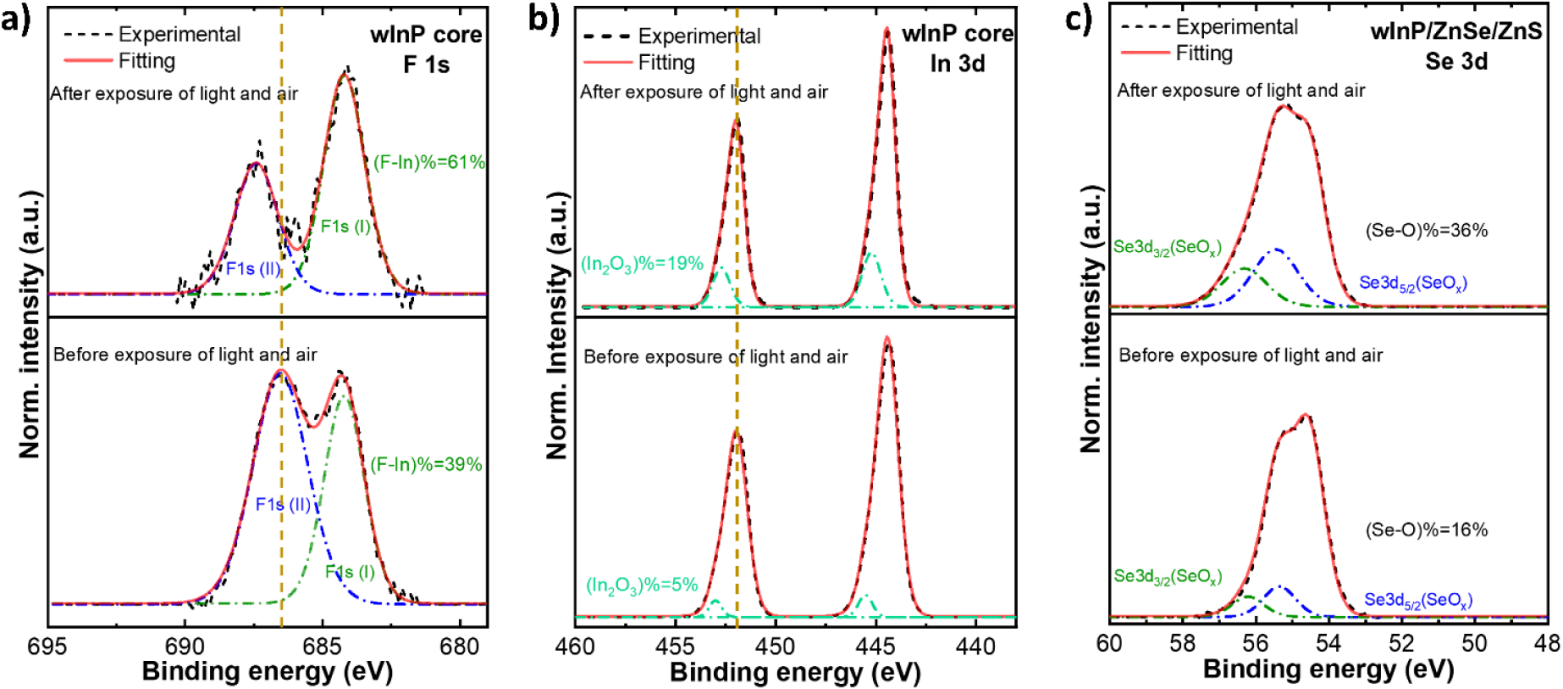
Surface analysis of w-InP and w-InP/ZnSe/ZnS core/shell QDs after 6 h of continuous illumination in an ambient environment (bottom and top panels are before and after photo-oxidation, respectively): (a–b) High-resolution XPS (HR-XPS) spectra (F 1s; In 3d) of w-InP core QDs, showing an increasing proportion of In–F and In–O species after being exposed to light and air (c) HR-XPS spectra (Se 3d) of w-InP/ZnSe/ZnS CSS QDs before and after photo-oxidation, showing an increasing proportion of Se–O species after being exposed to light and air (black short dashed line: experimental data; red solid line: envelope fitting; dot-dashed line: deconvoluted components).

In contrast, photoinduced surface oxidation of w-InP/ZnSe/ZnS QDs leads to significant PL quenching. XPS analysis of a sample after irradiation under ambient conditions shows an increase in Se–O species from 16% to 36%, clearly indicating photo-oxidation (Figure 5c). A minimal degree of interfacial oxidation may already be present after shell passivation due to the use of zinc carboxylates and oleylamine at an elevated temperature, which can promote amide formation and residual water release, as also reported in the literature on *zinc blende* InP-based core/shell systems.^51,52^ However, the pronounced increase in oxidized Se species upon illumination confirms that further oxidation is induced, showing that intense 405 nm LED light exposure in an ambient atmosphere may break Zn–Se bonds, thereby compromising shell integrity and diminishing its protective function over the w-InP core. This degradation promotes oxidation of the shell materials, leading to the formation of dangling bonds at the core/shell interface and possibly at the outer QD surface (note that the outer ZnS shell is thin).^53^ These defects act as nonradiative recombination centers, ultimately reducing the PL efficiency.

To gain further insight regarding the photo-oxidation mechanism, 6-h continuous illumination experiments were conducted in the presence of oxygen using incident wavelengths of 405 nm (3.1 eV, above the ZnSe band gap^54^), 532 nm (2.3 eV, below the ZnSe gap), and 650 nm (1.9 eV, below the ZnSe gap). The rationale behind this experiment is that 405 nm light has sufficient energy to excite electronic transitions that delocalize into the ZnSe shell region, while the longer wavelengths lack the energy to do so and therefore excite only transitions within the InP core. The corresponding PLQY decrease, plotted versus the number of absorbed photons (Figure 6a) shows that illumination at 405 nm induces the most rapid and significant PL decrease, while 532 nm and 650 nm result in significantly slower quenching, reaching a plateau of ∼40% PLQY while closely resembling the behavior observed under oxygen-free illumination in Figure 4c. These findings further highlight that the photodegradation is related to the ZnSe shell, as only 405 nm excitation provides sufficient energy to directly and continuously degrade and photo-oxidize this region, damaging its quality and generating new trap states at the core/shell interface and the outer surface, which leads to pronounced PL quenching. The results also suggest that these highly emissive CSS QDs are relatively stable and applicable even in ambient conditions in systems employing lower-photon-energy light sources, rather than blue light, to mitigate PL degradation.

**Figure 6:**
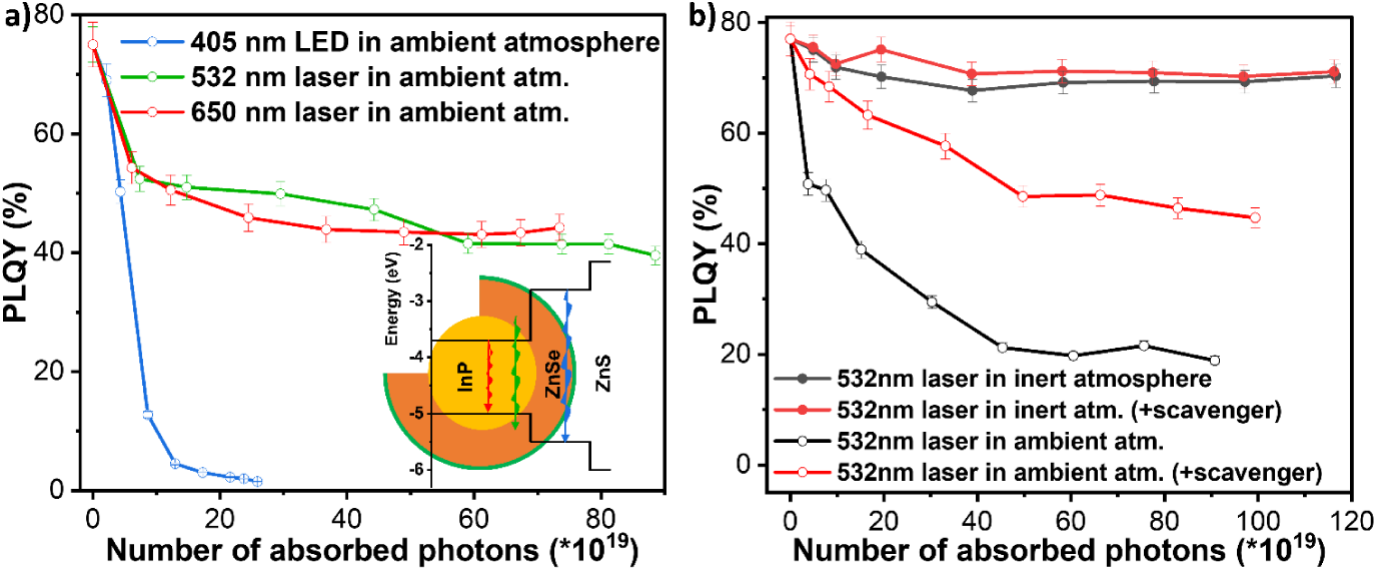
Mechanism study on photo-oxidation-induced PL degradation of w-InP/ZnSe/ZnS CSS QDs: (a) PL intensity variation of w-InP/ZnSe/ZnS CSS QDs after exposure to air and illumination, with different incident light wavelengths (405 nm blue LED, 532 nm green laser, and 650 nm red laser) (the energy level scheme of w-InP/ZnSe/ZnS QDs is attached) (b) PLQY decrease of w-InP/ZnSe/ZnS CSS QDs under 532 nm light and air, with and without the addition of a singlet-oxygen scavenger molecule (1,3-diphenylisobenzofuran) (the calculation of the number of absorbed photons, as well as absorption spectra of singlet oxygen scavenger molecules and tested samples, are provided in the SI).

Similar photodegradation trends are observed in prior work on *zinc blende* InP/ZnSe QDs, where PL decrease under 455 nm (∼2.73 eV, near the ZnSe band gap) illumination was attributed to a shallow valence band offset that facilitates hole leakage and promotes photo-oxidation; this phenomenon becomes more pronounced as particle size decreases due to further band offset reduction.^55,56^ This mechanism aligns with our observed PLQY degradation in *wurtzite* InP-based CSS QDs. Additionally, both studies highlight the challenges associated with outer ZnS passivation, suggesting that incomplete coverage over the vulnerable ZnSe shell leaves the structure susceptible to oxidative damage under illumination. These similarities underscore common limitations in InP/ZnSe heterostructures, irrespective of crystal phase, where band alignment, interface quality, and ZnSe surface reactivity play critical roles in governing long-term photostability.

To further comprehend the photo-oxidation mechanism in w-InP/ZnSe/ZnS QDs, a comparative experiment was conducted using 1,3-diphenylisobenzofuran (DPBF) as a singlet-oxygen scavenger.^57^ Figure 6b presents the PLQY versus the number of absorbed photons upon continuous 532 nm illumination in air. The presence of DPBF noticeably slowed the PL quenching, indicating that singlet oxygen contributes significantly to fluorescence degradation. Moreover, the observed decrease in DPBF absorbance at ∼410 nm confirms its consumption via reaction with singlet oxygen (Figure S9). These results support a mechanism in which excitons generated under illumination promote singlet-oxygen formation, thereby accelerating the ZnSe shell oxidation and inducing structural damage and trap formation.

To generalize the shell growth approach and demonstrate its applicability for tuning emission wavelengths in the NIR, we also applied a similar synthesis strategy to additional w-InP core sizes. Previously, we showed that Cu_3–*x*_P QDs can be size-controlled by adjusting the type of copper salt precursors, precursor concentration, and ligand composition, enabling the preparation of w-InP QDs with well-defined dimensions after cation exchange and NOBF_4_ treatment.^26^ Here, three representative QD sizes—8, 11, and 13 nm—are presented. Figure S10 shows the absorption spectra of Cu_3–*x*_P and corresponding cation-exchanged w-InP QDs, normalized at 500 nm. As reported, the localized surface plasmon resonance (LSPR) of Cu_3–*x*_P depends on NC size and copper vacancy concentration; within this size range, larger QDs exhibit stronger plasmonic absorption, while the LSPR peak remains broadly centered near 1400 nm with small size dependence.^23,26^ After cation exchange, the characteristic plasmonic absorption disappears, and a band-edge onset near 830 nm emerges, confirming the formation of w-InP. Subsequently, the post-synthetic NOBF_4_ treatment was performed for 1 h. This step yields well-resolved excitonic absorption peaks and measurable PL, as shown in Figure 7a and Figure S11. After NOBF_4_ treatment, the core sizes are reduced to 6.8, 8.7, and 12.0 nm, and exhibit band gap excitonic absorption peaks at ∼740, ∼780, and ∼820 nm, respectively, with corresponding PL showing small Stokes shifts and narrow line-widths. Both absorption and PL redshift systematically with increasing core size, consistent with the quantum confinement effect.

**Figure 7:**
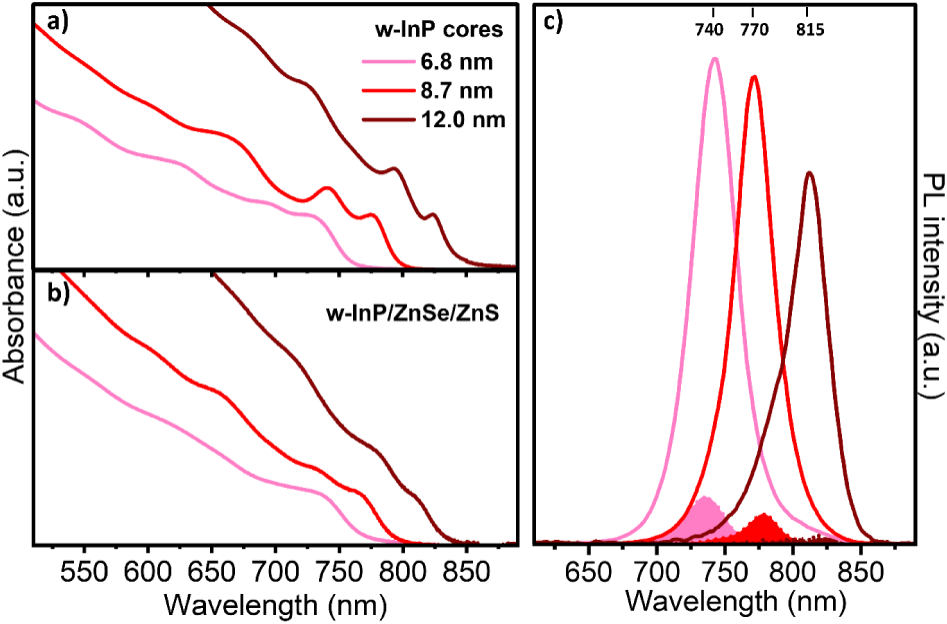
(a–b) Absorption spectra of w-InP cores (top) and the corresponding w-InP/ZnSe/ZnS CSS QDs (bottom) with core sizes of 6.8 nm (pink), 8.7 nm (red), and 12.0 nm (brown). (c) PL spectra of the w-InP cores and the corresponding CSS QDs, showing a size-tuned red shift from 740 to 820 nm, accompanied by enhanced PL intensity and preservation of a narrow fwhm after shell growth.

TEM analysis (Figure S12) further confirms the size and morphology of the Cu_3–*x*_P templates, cation-exchanged w-InP, and NOBF_4_-treated w-InP QDs. Cation exchange produces only a slight size reduction, which is attributed to the retention of the anion sublattice and modest lattice contraction from Cu_3–*x*_P to w-InP. A further small decrease after NOBF_4_ treatment reflects the removal of surface copper residues and oxide impurities.

It has been previously reported that NOBF_4_-treated bare w-InP cores can achieve a PLQY of ∼30% after several hours at room temperature and up to ∼40% after ∼1 h at 50 °C.^26^ However, such improvements are accompanied by significant additional changes in the optical properties due to the surface etching reaction. These changes include strongly smeared absorption features, pronounced PL broadening, and a substantial blue shift. Here, a similar surface reaction with an extended duration is performed on the most recent, larger w-InP core sample (Figure S13). After 24 h of NOBF4 treatment at room temperature, the PLQY reaches ∼20%, while the excitonic absorption features become poorly resolved, the emission undergoes a blue shift from ∼820 to ∼760 nm, and the PL fwhm increases from ∼40 to ∼90 meV. These results highlight the limitations of prolonged surface etching. Therefore, rather than pursuing extended surface treatment, epitaxial shell passivation is developed herein, which preserves well-defined optical characteristics, maintains narrow emission line widths, and enables access to deeper near-infrared (NIR) emission (i.e., beyond 800 nm), showcasing the advantages of this approach.

Identical ZnSe/ZnS dual-shell growth conditions were applied to w-InP cores emitting at ∼740 nm (6.8 ± 0.7 nm) and ∼820 nm (12.0 ± 0.9 nm), extending the approach beyond the ∼780 nm (8.7 ± 0.7 nm) sample discussed above. The absorption features of the resulting CSS QDs are shown in Figure 7b. Following shell growth, the first excitonic absorption peaks exhibit only minimal shifts, appearing at ∼740, ∼770, and ∼815 nm, respectively. The negligible change in transition energy indicates that both charge carriers remain largely confined within the core, with limited wave function delocalization into the shell. In addition, the PL efficiency increases substantially across all core sizes while preserving narrow emission line widths (Figure 7c), consistent with effective surface passivation that suppresses nonradiative recombination without significantly altering the electronic structure.

Shell passivation leads to a pronounced enhancement in PLQY across all core sizes. As discussed in the previous section, cores emitting at ∼780 nm exhibit an increase in PLQY from ∼5% to ∼78%, while the ∼740 nm sample improves from ∼8% to ∼81%. Even for the largest cores (∼820 nm), where surface-related losses are more severe, the PLQY rises substantially from ∼2% to ∼62%. Importantly, these enhancements follow the previously established correlation between the initial core PLQY and the final PLQY after shell growth (Figure 2a).

Under comparable shell thickness, the present size series adheres to the same trend, indicating that improved interfacial quality facilitates more effective and uniform shell nucleation and growth, thereby promoting a higher radiative efficiency. The consistent enhancement observed across the entire size range underscores the effectiveness of the dual-shell architecture in suppressing nonradiative recombination over an extensive quantum-confined regime. In addition, all CSS QDs maintain narrow emission line widths (fwhm = 33–38 nm, <90 meV), reflecting a uniform size distribution and minimal inhomogeneous broadening.

The optical and structural characterization of CSS QDs derived from 6.8 nm cores is presented in Figures S14-S16. Deposition of 3.8 MLs of ZnSe followed by 1.2 ML of ZnS increases the PLQY to ∼81% and modestly extends the average lifetime (τ_avg_) from 49 to 56 ns (Figure S14, Table S4). The absolute increase in τ_avg_ is less significant compared to the larger-core sample. This arises because the smaller-core QDs already exhibit a relatively long τ_avg_ (49 ns), indicating an initially higher fraction of bright particles. Consequently, shelling induces only a modest increase in τ_avg_ (to 56 ns). In contrast, the larger-core sample contains a greater fraction of dark particles before shelling, which leads to a substantial enhancement in τ_avg_ (36 → 66 ns) upon effective shell passivation. Overall, the pronounced enhancement of PLQY can be attributed mostly to the activation of previously dark particles by the shell, leading to a substantially larger fraction of radiatively recombining QDs. HAADF-STEM imaging and EDX elemental mapping (Figure S15) confirm uniform shell coverage and effective surface passivation, consistent with observations for larger-core counterparts. Polarization-resolved PLE measurements (Figure S16) show clear luminescence anisotropy in both core-only and CSS QDs, confirming the preservation of the lower-symmetry wurtzite crystal structure after shell growth.

Furthermore, extending this gradient-shell-growth strategy to the weak-confinement regime, we aim to achieve deeper NIR emission, a challenging regime for InP QDs due to limitations in synthesizing large cores via traditional hot-injection methods and the prevalence of trap-mediated nonradiative losses. Using the same epitaxial shelling approach, w-InP/ZnSe/ZnS QDs emitting at ∼815 nm are obtained with a PLQY of ∼62% and a narrow fwhm of ∼35 nm (∼65 meV) (Figure S17), extending InP emission into the deep NIR while preserving high spectral purity. The corresponding large-core w-InP precursors exhibit very low initial PLQY (1–2%), even after NOBF_4_ treatment (50 °C, 15 min), reflecting a high density of surface traps. Shell growth activates these dark states, leading to a dramatic increase in τ_avg_ (29 → 105 ns, Table S5). The pronounced lifetime extension also suggests partial electron delocalization into the shell, consistent with quasi-type-II behavior in the weak-confinement regime. Although EDX mapping (Figure S18) shows less uniform shell coverage, likely due to reduced core curvature and increased lattice strain, the sustained high PLQY at 815 nm demonstrates that this dual-shell architecture effectively suppresses nonradiative recombination, providing a novel and robust pathway to effective deep-NIR-emissive InP-based nanomaterials.

## Conclusions

We report the synthesis of high-quality w-InP/ZnSe/ZnS CSS QDs, which exhibit intense near-infrared (740–820 nm) fluorescence with a high PLQY of 60–80% and a narrow fwhm (33–38 nm, <90 meV). The optical performance of the CSS QDs based on different core sizes—including emission peak position, PLQY, and fwhm—along with the comparison to previously reported works on NIR-based InP, is summarized in Table 1.

**Table 1:** Comparison of Optical Properties of Previously Reported Results with This Work

Journal	Emission (nm)	PLQY (%)	fwhm (nm)
**This work**	740	81	38
	780	78	33
	815	62	35
Chemical Engineering Journal, Volume 525, 2025, 170430 ^58^	683–742	40–74	64–57
Small 2024, 20, 2404426 ^59^	725	42.5	45
J. Am. Chem. Soc. 2023, 145, 10, 5970–5981 ^22^	728	–	48
Laser Photonics Rev 2024, 18, 2400367 ^40^	807	6–12	111

The outstanding optical performance was achieved through a study of targeted parameters in the synthesis. This included studies on improving the surface quality of w-InP cores via the NOBF_4_ surface reaction to facilitate more efficient and defect-free shell growth, increasing the ZnSe shell growth temperature to enhance crystallinity and uniformity, and introducing an intermediate purification step prior to ZnS shell growth to remove unreacted precursors, excess ligands, and byproducts, thereby enabling more controlled shell deposition. Moreover, we conducted a photostability study to evaluate the long-term optical performance of the QDs under various conditions. Both w-InP cores and w-InP/ZnSe/ZnS QDs demonstrated remarkable stability against oxidation in the absence of light. Interestingly, under photoirradiation, w-InP cores showed a notable increase in PLQY, more so in ambient atmosphere. This enhancement was attributed to the formation of thin In–O and In–F layers, which effectively reduced nonradiative recombination and enhanced PL efficiency. On the other hand, the PL performance of the CSS QDs degraded under continuous illumination. This decline is primarily linked to photo-oxidation processes occurring mainly in the ZnSe shell, which compromised the integrity of the core/shell interface and outer surface and caused exciton quenching. However, this degradation was mitigated when the QDs were exposed to lower-energy light sources (e.g., green or red lasers), as compared to higher-energy illumination that directly excites the ZnSe shell region (i.e., blue LEDs), due to better preservation of shell integrity.

Overall, this w-InP-based QD system addresses several longstanding challenges in technologically relevant InP-based QD synthesis, particularly in achieving NIR emission with high PLQY, narrow size distribution, and enhanced photostability. These findings support the advancement of Cd-free QDs for optoelectronic applications and provide insights into strategies for improving their long-term stability.

## Materials and Methods

### Materials

Oleylamine (OLA, 80–90%) was purchased from Thermo Scientific. 1-Octadecene (1-ODE, 90%), squalane (SQ, 95%), oleic acid (OA, 90%), trioctylphosphine (TOP, 97%), triphenyl phosphite (TPOP, 97%), diphenyl phosphide (DPP, 97%), copper acetate (CuAc_2_, 99.99%), copper tartrate hydrate (CuTar_2_, 99.9%), indium­(III) bromide (InBr_3_, 99.999%), nitrosyl tetrafluoroborate (NOBF_4_, 95%), zinc­(II) fluoride (ZnF_2_, 99.99%), zinc oxide (ZnO, 99.99%), selenium powder (Se, 99.99%), and 1-octanethiol (1-OTT, 98.5%) were purchased from Sigma-Aldrich. Toluene and ethanol (analytical grade) were obtained from various commercial suppliers. All reagents and solvents were used without further purification.

### Instrumentation

Absorption spectra were acquired using a Jasco V-570 UV–vis–NIR spectrophotometer. PL spectra were measured with a Cary Eclipse spectrofluorometer, and PLQY was measured using a Hamamatsu Absolute PL Quantum Yield spectrometer C11347-11. Time-resolved PL and anisotropy measurements were conducted on an Edinburgh Instruments FL920. Transmission electron microscopy (TEM) imaging was performed on a Tecnai G2 Spirit Twin T12 microscope (Thermo Fisher Scientific) operated at 120 kV. High-resolution scanning-transmission electron microscopy (HR-STEM) and elemental mapping were carried out using a Themis Z aberration-corrected STEM (Thermo Fisher Scientific) at 300 kV, equipped with a high-angle annular dark-field (HAADF) detector and Super-X energy-dispersive X-ray spectroscopy (EDX) system; data acquisition and analysis were done using Velox software (Thermo Fisher Scientific). Powder X-ray diffraction (XRD) patterns were recorded using a Philips PW 1830/40 diffractometer equipped with Cu Kα radiation. X-ray photoelectron spectroscopy (XPS) was conducted on a Kratos Axis Supra with monochromatic Al Kα.

### Hexagonal Copper Phosphide Synthesis

Cu_3–*x*_P QDs were synthesized by a heat-up method using copper salts (CuTar_2_ or CuAc_2_), TPOP, and OLA under an inert atmosphere, based on an earlier report.^24^ For the synthesis of medium-sized Cu_3‑x_P particles (∼11 nm), 212 mg (1 mmol) of CuTar_2_ was mixed with 3.5 mL of OLA, 3.5 mL of TPOP, and 35 mL of 1-ODE. The mixture was degassed under vacuum at 50 °C for 30 min, then heated to 150 °C under argon and maintained at that temperature for 10 min. The temperature was subsequently raised to 280 °C, and once the target temperature was reached, the heating mantle was removed, and the solution was allowed to cool naturally. Decreasing the amount of 1-ODE, namely to increase the concentration of copper salt, allowed the formation of larger Cu_3–*x*_P QDs (>13 nm). For Cu_3–*x*_P QDs with small sizes (∼8 nm), 180 mg (1 mmol) of CuAc_2_ was mixed with 3.4 mL of OLA, 3.2 mL of TPOP, and 24 mL of 1-ODE, and the same procedure was followed. The crude Cu_3–*x*_P QDs were purified twice with toluene and ethanol as the solvent and antisolvent, respectively. The Cu_3–*x*_P QDs tend to oxidize within a few days under ambient conditions; thus, it is recommended to keep the purified QDs in the glovebox.

### Wurtzite Indium Phosphide Cores Preparation

w-InP QDs were synthesized through a cation exchange reaction, exchanging Cu^+^ with In^3+^ on hexagonal Cu_3–*x*_P QDs, based on prior reports.^23,25–27^ In a typical reaction, 2 mmol of InBr_3_, TOP (10 mL), and 1-ODE (10 mL) were mixed and degassed at 120 °C for 30 min, followed by heating to 200 °C under an argon flow. Once the reaction mixture reached 200 °C, the purified Cu_3–*x*_P QDs dispersed in 0.5 mL of toluene were injected into the reaction flask and reacted for 60 min at 200 °C. After the reaction, the solution was cooled, and the QDs were purified by precipitation with ethanol and redispersion in toluene. The final w-InP QDs were dispersed in 2 mL of toluene. Then, the cation-exchanged w-InP QDs were surface-reacted using a 1 M NOBF_4_-DMF solution.^25,26,60,61^ In this step, 1 mL of w-InP QDs in toluene (OD = 10 at the first excitonic absorption peak) was added to 10 mL of NOBF_4_ solution and kept at room temperature for 60 min. After the reaction, the QDs were precipitated by adding toluene, then redispersed in toluene and 2%_(v/v)_ OLA. The samples were stored in a glovebox for further experiments and characterizations.

### Shell Precursors Preparation

#### Preparation of 0.3 M High Reactivity Zinc Oleate (ZnOA_2_-H) Solution

9 mmol of ZnO and 8 mL of OA (25 mmol) were mixed and degassed at 120 °C for 60 min. The mixture was then heated under an argon flow to 300 °C and maintained at this temperature for 60 min. After that, the solution was allowed to cool naturally to 80 °C. At this point, 8 mL of OLA and 14 mL of 1-ODE were added to the solution. The mixture was degassed at 150 °C for 30 min and then stored in the glovebox.

#### Preparation of 0.1 M Low Reactivity Zinc Oleate (ZnOA_2_-L) Solution

2 mmol of ZnO and 6.4 mL of OA (20 mmol) were mixed and degassed at 120 °C for 60 min, heated under an argon flow to 300 °C, and kept for 60 min. Then, the solution was cooled down naturally to 80 °C; 6 mL of OLA and 16 mL of 1-ODE were added to the flask, and the mixture was degassed at 150 °C for 30 min and then stored in the glovebox.

#### Preparation of 0.2 M Se-TOP Solution

158 mg (2 mmol) selenium powder was mixed with TOP (5 mL) and 1-ODE (5 mL), along with the addition of 250 μL of DPP (2 mmol). The mixture was stirred overnight in the glovebox for further use.

#### Preparation of 0.13 M 1-OTT Solution

204 μL of 1-OTT (1.14 mmol) was mixed with 1-ODE (8.8 mL) and stored in a glovebox for further use.

### W-InP/ZnSe/ZnS Core/Shell QD Synthesis

To synthesize w-InP/ZnSe/ZnS QDs, 10 mg of ZnF_2_ and 10 mg of ZnSt_2_ ^15,45^ were mixed with 1 mL of OLA and 2 mL of SQ in a four-neck flask. The mixture was degassed at 80 °C for 60 min, and then heated to 340 °C under an argon flow. When the temperature reached 180 °C, 20 nmol of purified w-InP core QDs (after surface fluorination processing) were injected dropwise. Upon reaching 340 °C, 0.3 mL of high-reactivity zinc oleate (ZnOA_2_-H, 0.9 mmol) was injected rapidly, followed by the slow addition of 0.35 mL of Se-TOP solution (0.07 mmol) via a syringe pump at a rate of 1.4 mL h^–1^ for 15 min. After the addition, the reaction was maintained for an additional 5 min. Subsequently, 0.15 mL of OA and 0.15 mL of TOP were added, and the reaction mixture was transferred to a glovebox and purified using ethanol as an antisolvent for the next step. In a separate flask, 1.5 mL each of OLA, OA, and 1-ODE were mixed, degassed at 90 °C for 60 min, and then heated under argon to 310 °C. When the temperature reached 180 °C, the previously synthesized w-InP/ZnSe QDs were introduced. At 240 °C, 0.10 mmol of low-reactivity zinc oleate (ZnOA_2_-L) and 0.13 mmol of 1-octanethiol (1-OTT) were simultaneously added via a syringe pump. The reaction was maintained for 60 min and held for an additional 5 min before cooling. The resulting w-InP/ZnSe/ZnS QDs were purified in the glovebox using ethanol as an antisolvent and stored for further characterization and experiments.

### Photostability Study

The w-InP cores and w-InP/ZnSe/ZnS QD samples (each with a concentration of 2 nmol/mL), dispersed in toluene, were placed in quartz cuvettes and subjected to four specific conditions for 6 h: [1] dark without air, [2] dark with air, [3] continuous exposure to a 6 mW 405 nm blue LED under ambient air, and [4] exposure to the same LED under an inert atmosphere. Fluorescence intensity was monitored, and the variation in PLQY over time was used to evaluate photostability.

For wavelength-dependent experiments, the samples were exposed to three light sources: a 6 mW 405 nm blue LED, a 30 mW 532 nm green laser, and a 30 mW 650 nm red laser, in air for 6 h. The PLQY values were recorded at regular intervals.

For the mechanism study, the singlet-oxygen scavenger experiment was performed.^57^ 1,3-Diphenylisobenzofuran (DPBF) was added to the QD samples at a molar ratio of 1.5:1 (DPBF in excess), with a control sample prepared without the scavenger. PLQY and absorption spectra were monitored during exposure.

## Supplementary Material



## Notes

Characteristics of Cu_3‑x_P and cation-exchanged w-InP QDs; TRPL results and line-fitting of w-InP cores and w-InP/ZnSe/ZnS QDs; STEM-EDS line scan, elemental mappings, and HR-STEM images of the w-InP/ZnSe/ZnS QDs; luminescent anisotropic characterization of w-InP and w-InP/ZnSe/ZnS QDs; optical and structural characteristics of w-InP/ZnSe/ZnS QDs synthesized based on the other two core sizes (PDF)
