# Supplementary material for: Wurtzite InP/ZnSe/ZnS Core/Shell Semiconductor Quantum Dots with Bright Near-IR Emission
Source: J Am Chem Soc. 2026 Jun 15;148(25):26092–104. doi: 10.1021/jacs.6c05042 (PMC13339127; doi:10.1021/jacs.6c05042)
Supplement: Supplementary file 1 [file ja6c05042_si_001.pdf]

Supporting Information

Wurtzite InP/ZnSe/ZnS Core/shell Semiconductor Quantum Dots with Bright Near-IR Emission

Jiekai Dai[†‡], David Stone[†‡], Xiang Li[†‡], Adar Levi[†‡], Sergei Remennik[‡] and Uri Banin[*†‡]

[†]Institute of Chemistry, The Hebrew University of Jerusalem, Jerusalem 91904, Israel

[‡]The Center for Nanoscience and Nanotechnology, The Hebrew University of Jerusalem, Jerusalem 91904, Israel

*E-mail: uri.banin@mail.huji.ac.il

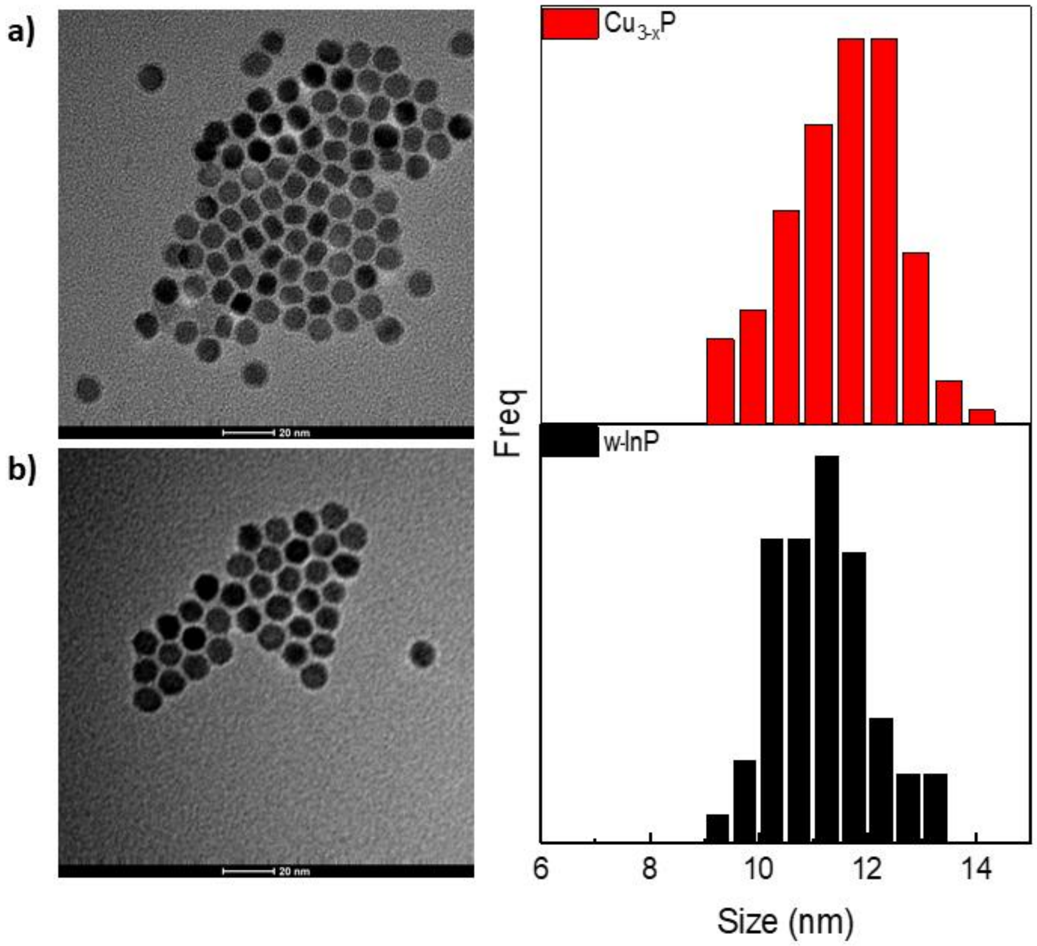

Figure S1. TEM images and corresponding diameter histograms of (a) hexagonal $Cu_{3-x}P$ and (b) cation-exchanged w-InP quantum dots (QDs)

Time Resolved Photo-luminescence Analysis

The PL decay was fitted with bi-exponential decay functions ($I(t) = A_1 e^{-t/\tau_1} + A_2 e^{-t/\tau_2}$), where A_i and τ_i represent the amplitude and lifetime of the i-th decay component, respectively. To quantify the overall emission response, an intensity-weighted average lifetime was used:

$$\tau_{avg} = \frac{A_1\tau_1^2 + A_2\tau_2^2}{A_1\tau_1 + A_2\tau_2}$$

Our study shows that the kinetics are **inhomogeneous**, meaning that the emission generally arises from distinct emissive subpopulations rather than from a single species undergoing competitive decay pathways. Therefore, it is also possible that each decay component corresponds to a subpopulation with its own characteristic microenvironment and radiative / nonradiative decay rates.

Table S1. Summary of parameters in terms of time resolved photo-luminescence (TRPL) spectra for w-InP cores (d ~ 8.7 nm) and resulting core/shell/shell (CSS) QDs (using bi-exponential fitting model)

	PLQY (%)	τ_1 (ns)	A_1	τ_2 (ns)	A_2	τ_{avg} (ns)
w-InP core	5	8.82	0.709	47.8	0.291	36
w-InP/ZnSe/ZnS	78	14.5	0.465	74.5	0.535	66

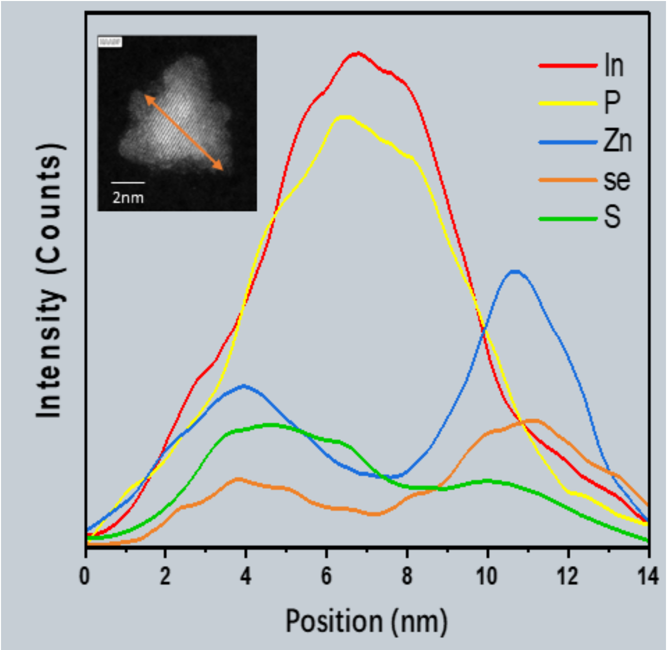

Figure S2. Energy dispersive X-ray (EDX) line scanning of a single w-InP/ZnSe/ZnS QDs in terms of the elements In, P, Zn, Se and S.

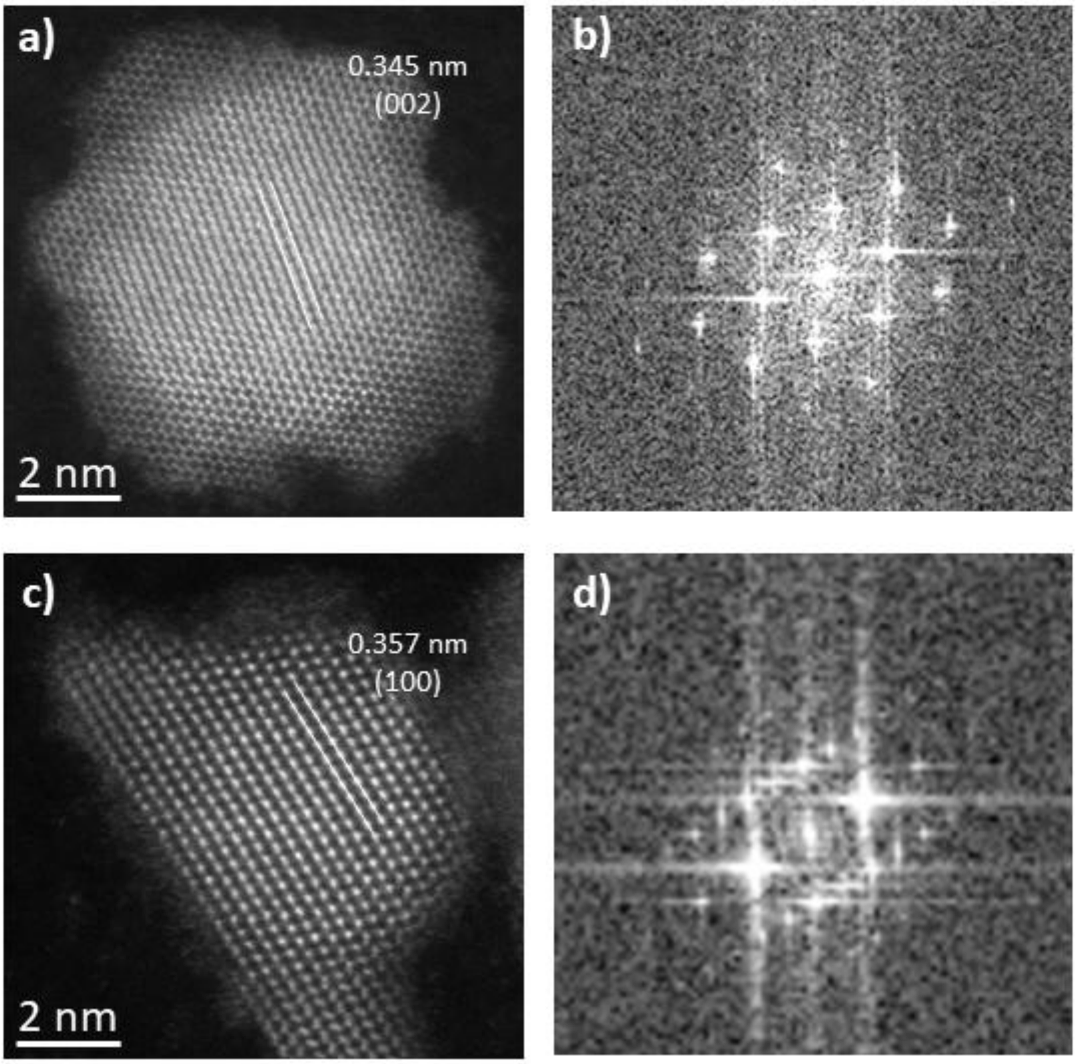

Figure S3. High-resolution HAADF-STEM analysis of single w-InP/ZnSe/ZnS CSS QDs: (a-b) (002) hexagonal basal facet and corresponding FFT. (c-d) (100) prism facet and FFT. The core size was 8.7±0.7 nm with ZnSe shell thickness of 3.5 MLs and ZnS outer shell of 1.2 ML

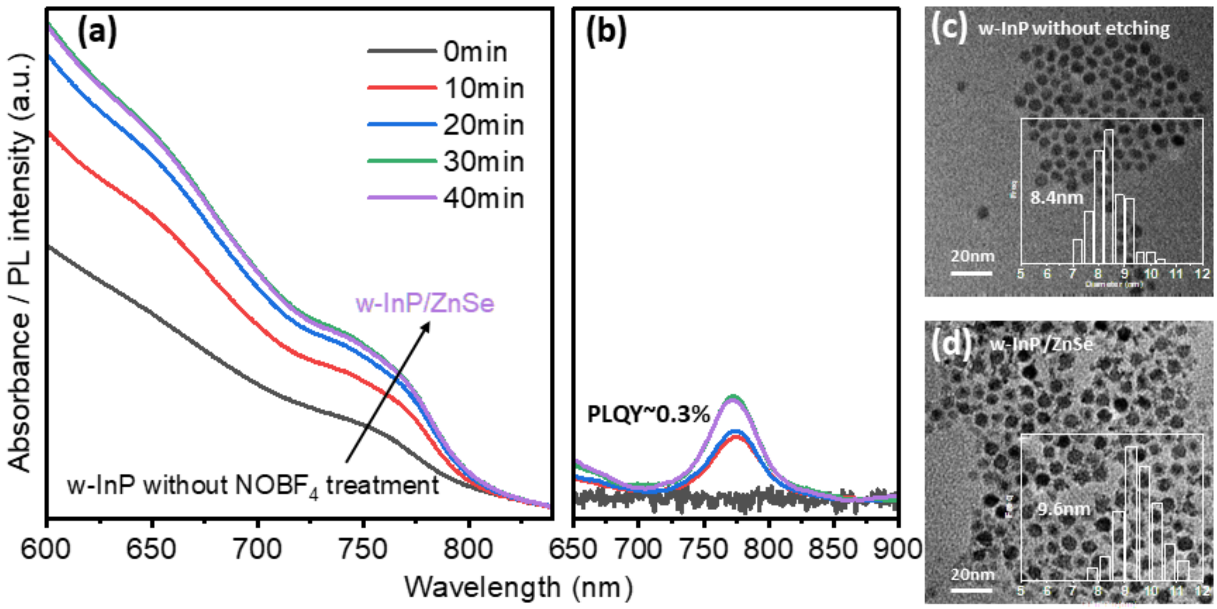

Figure S4. Growing shell on cation-exchanged w-InP **without** $NOBF_4$ reaction: (a-b) Absorption and PL spectra of the shell passivation progress (c-d) TEM images and corresponding diameter histograms of as-synthesized w-InP and w-InP/ZnSe.

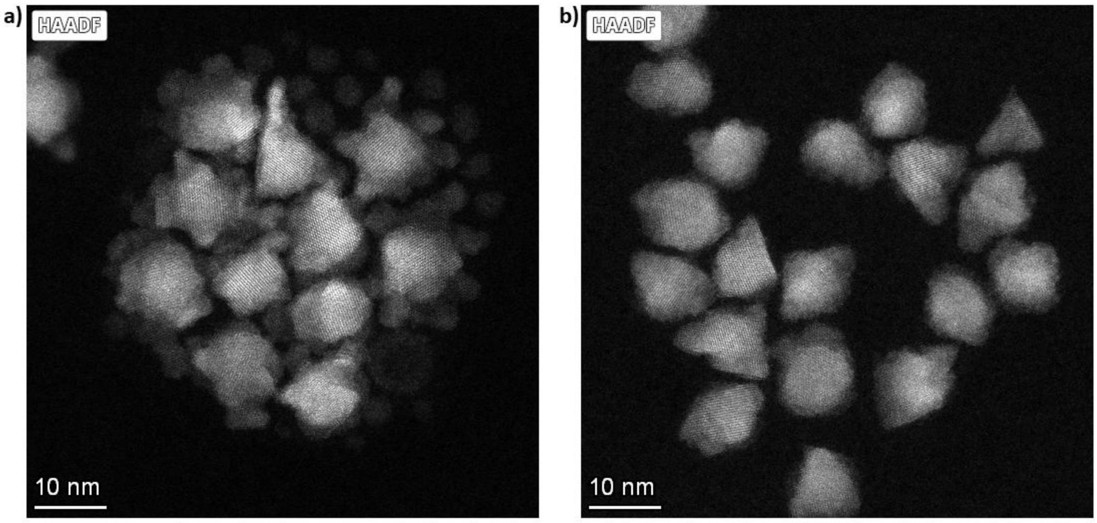

Figure S5. High resolution HAADF-STEM image of w-InP/ZnSe/ZnS QDs (a) without and (b) with intermediate purification step before outer ZnS shell growth

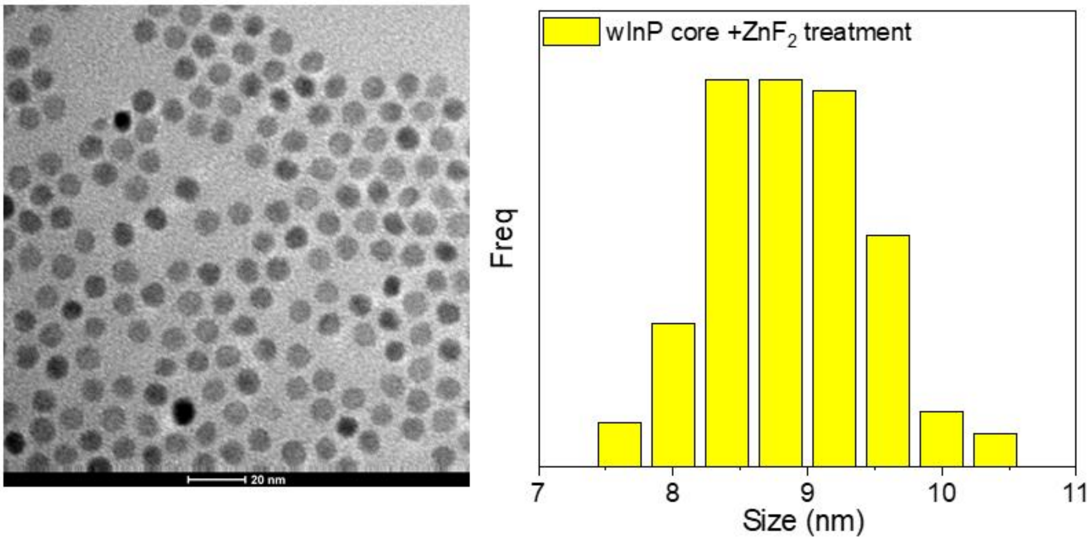

Figure S6. TEM image and corresponding diameter histogram of w-InP core QDs after ZnF_2 in-situ surface treatment

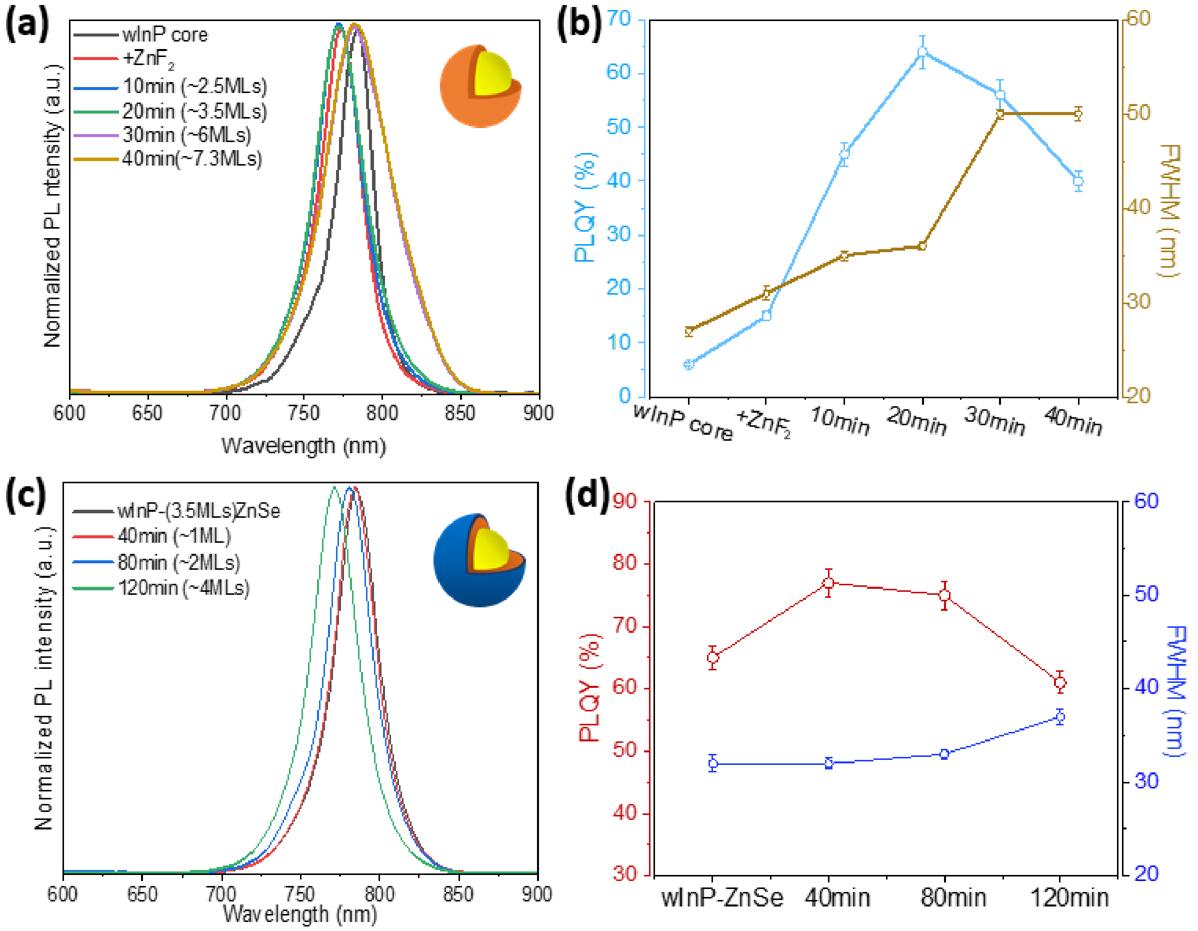

Figure S7. The effect of thickness of inner ZnSe shell and outer ZnS shell regarding to PL: (a) Normalized PL spectra of QDs during ZnSe shell growth with different thicknesses (~2.5 to ~7.3 MLs) (b) PLQY measurements and corresponding fwhm during ZnSe shell growth (c) Starting with optimized **3.5MLs** of ZnSe shell, normalized PL spectra of QDs after outer ZnS shell growth with 1–4 monolayers. (d) PLQY and fwhm with increasing ZnS shell thickness

Table S2. Summary of parameters in terms of TRPL spectra for w-InP cores (d~**8.7** nm) before and after exposure of continuous illumination in ambient environment (using bi-exponential fitting model)

	PLQY (%)	τ_1 (ns)	A_1	τ_2 (ns)	A_2	τ_{avg} (ns)
0	5	11.0	0.637	55.4	0.363	44
6hr	19	13.0	0.589	57.5	0.411	47

Table S3. Summary of parameters in terms of TRPL spectra for resulting w-InP/ZnSe/ZnS (based on **8.7nm** cores) before and after exposure of light in the presence of air (using bi-exponential fitting model)

	PLQY (%)	τ_1 (ns)	A_1	τ_2 (ns)	A_2	τ_{avg} (ns)
0	78	14.5	0.465	74.5	0.535	66
6hr	1.5	9.72	0.728	54.5	0.272	40

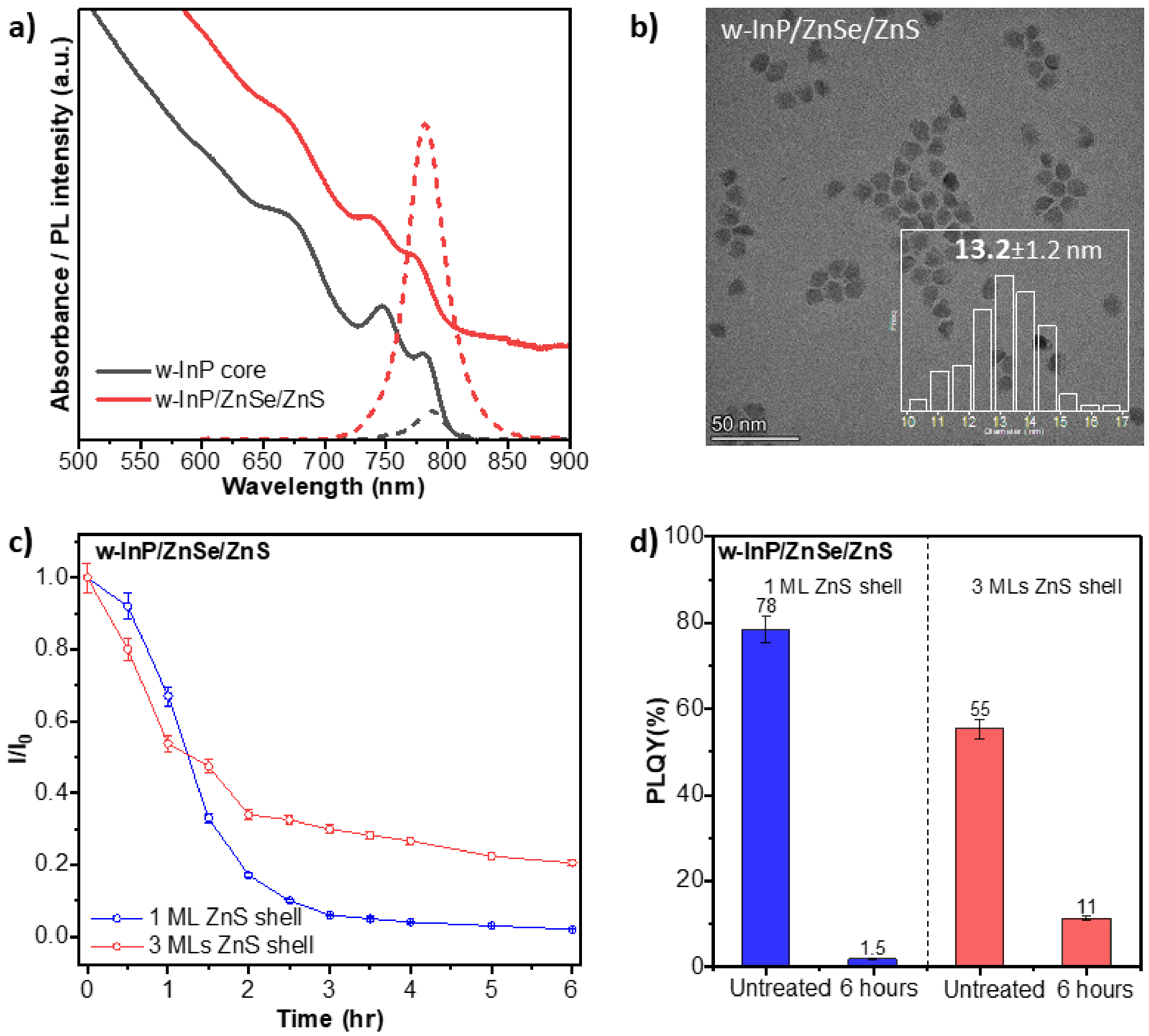

Figure S8. (a) Absorption and emission spectra of w-InP core (8.7±0.7 nm) and corresponding core/shell/shell QDs (b) TEM image of w-InP/ZnSe/ZnS QDs with 3.6MLs of ZnSe shell and 3 MLs of ZnS shell (c) PL change versus time under conditions of ambient+light (405 nm, 3 mW), for w-InP/ZnSe/ZnS QDs with two different ZnS outer shell thickness. (d) PLQY value of w-InP/ZnSe/ZnS QDs with two different ZnS outer shell thickness before and after exposure to blue light and air.

Calculation on number of absorbed photons based on the incident light sources and optical density (O.D.) of tested samples

$$n = \frac{Q}{\epsilon} = \frac{P(W) \cdot \%Abs}{\frac{(1240 \cdot 1.60 \cdot 10^{-19}) J \cdot nm}{\lambda_l \ (nm)}}$$

Where:

n: Number of photons absorbed per unit time (s^{-1});

Q: Effective power of the incident light to the tested sample (W);

ϵ: Energy of a single photon (J);

P: Power of the light source (W)

%Abs: Percentage of photons absorbed by the sample

λ_l: Wavelength of incident light (nm)

Number of absorbed photons during incident-wavelength-dependent photostability test according to the upper equation:

λ_l (nm)	O.D.	%Abs	P (mW)	n ($* 10^{16}/s$)
405	1.74	0.98	6	1.2
532	0.31	0.51	30	4.1
650	0.19	0.35	30	3.5

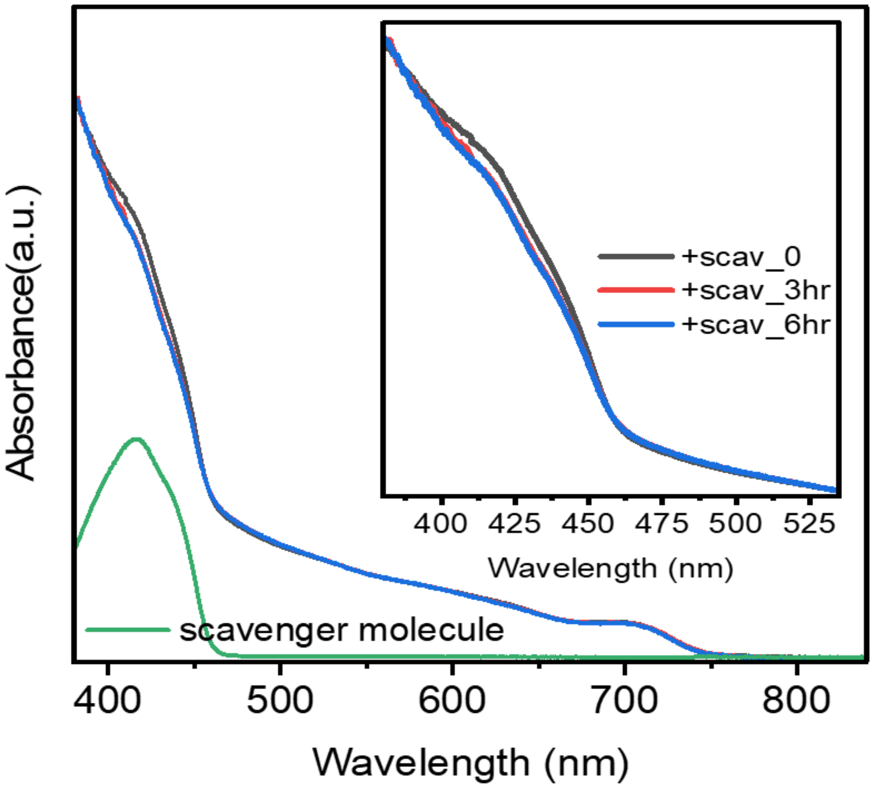

Figure S9. Absorption spectra of 1,3-diphenylisobenzofuran (DPBF) as singlet oxygen scavenger molecules, and of w-InP/ZnSe/ZnS with addition of scavenger molecules after 3 and 6 hours of illumination (with **532 nm** laser) in air

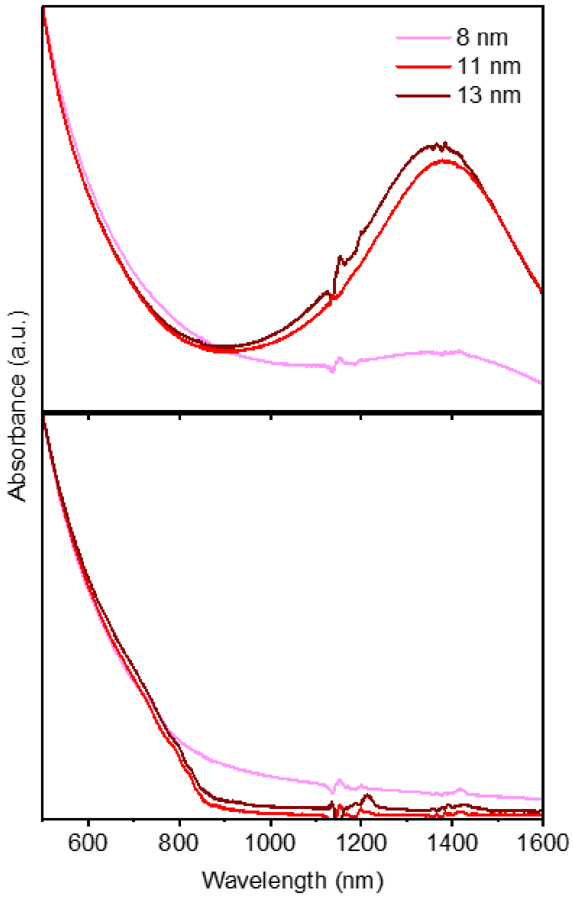

Figure S10. Absorption spectra of hexagonal $Cu_{3-x}P$ (top) and cation-exchanged w-InP (bottom) with different average diameters (pink: d~8 nm; red: d~11 nm; brown: d~13nm)

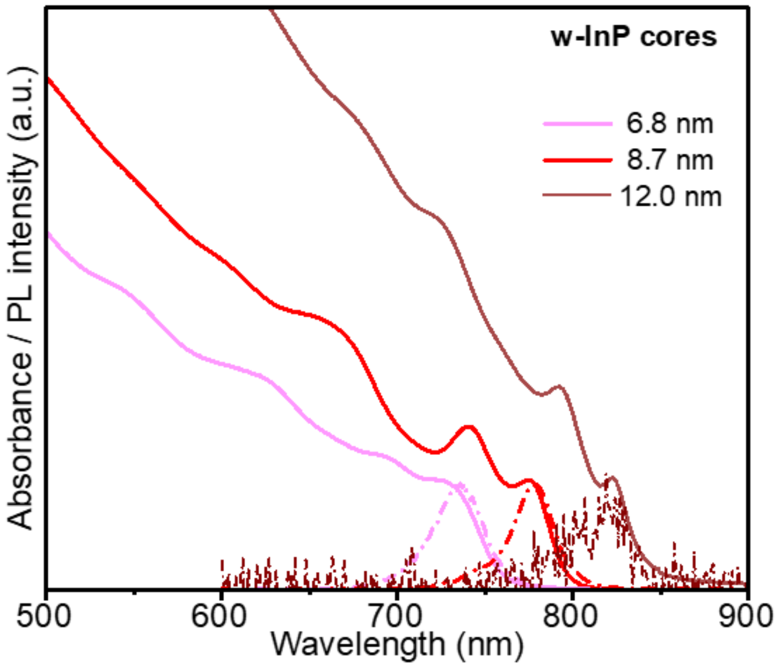

Figure S11. Absorption (solid line) and normalized PL (dash-dot line) spectra of 1hr-$NOBF_4$-etched w-InP core QDs with different average diameters (pink: d~8 nm; red: d~11 nm; brown: d~13nm)

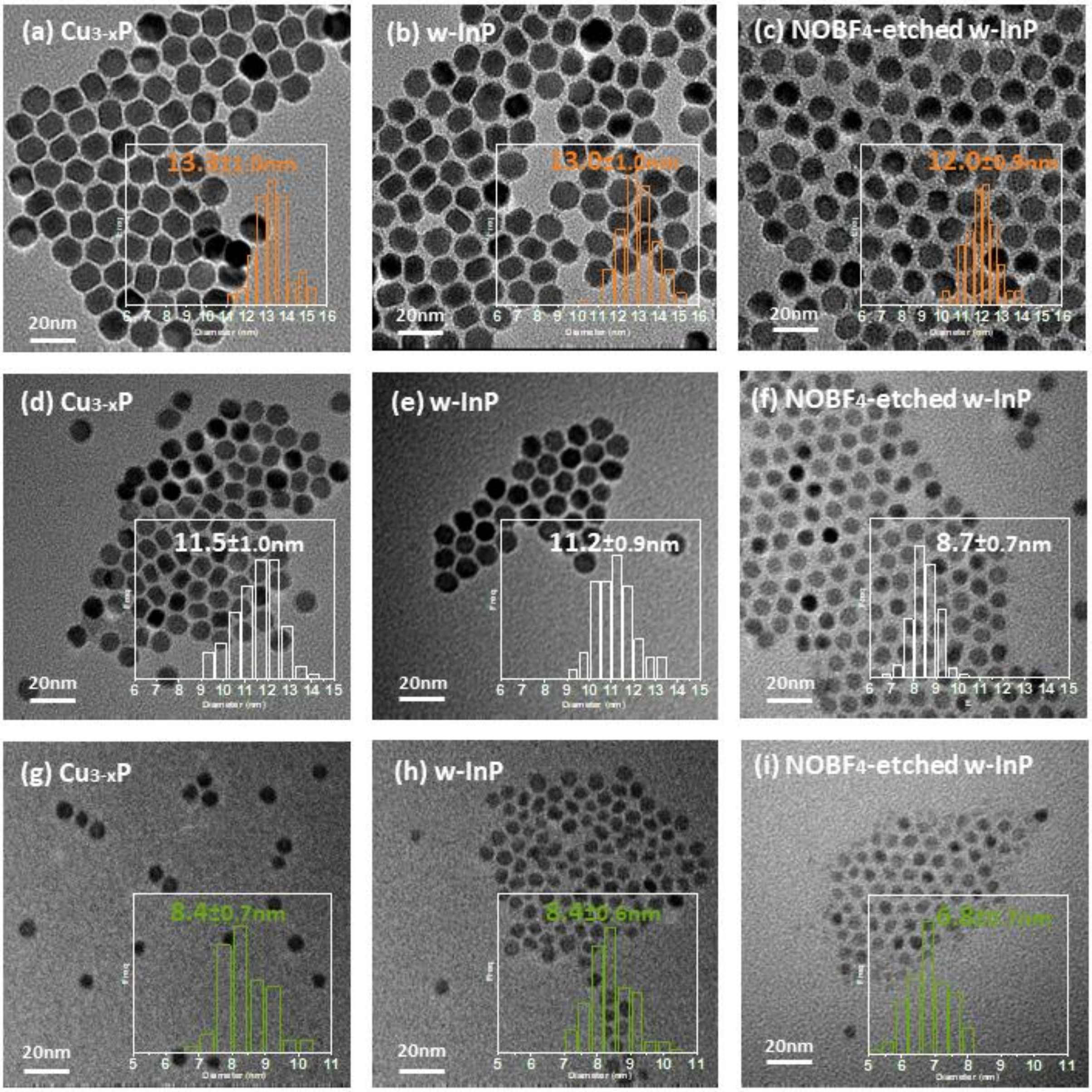

Figure S12. TEM images and corresponding diameter histograms of $Cu_{3-x}P$, cation-exchanged w-InP, and $NOBF_4$-treated w-InP QDs, for three different initial particle sizes. (a–c) Starting with $Cu_{3-x}P$ QDs of 13.3±1.0 nm average diameter, resulting in w-InP cores of 12.0±0.9 nm after exchange and etching. (d–f) Starting with $Cu_{3-x}P$ QDs of 11.5±1.0 nm, resulting in w-InP cores of 8.7±0.7 nm. (g–i) Starting with $Cu_{3-x}P$ QDs of 8.4±0.7 nm, resulting in w-InP cores of 6.8±0.7 nm.

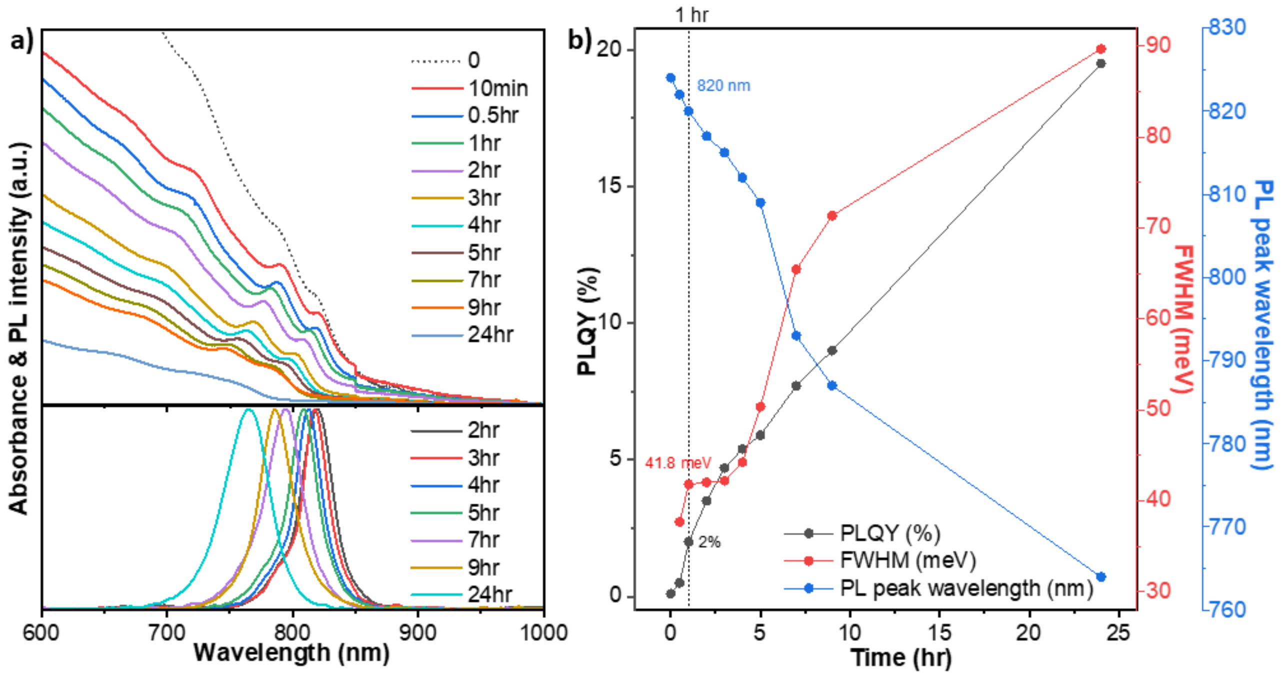

Figure S13. w-InP QDs treated with $NOBF_4$ at room temperature: (a) absorption (above) and emission (below) spectra of w-InP measured during the surface reaction with $NOBF_4$. (b) Change on PLQY (left axis, black dots and line), PL fwhm (right axis, red dots and line) and PL peak wavelength (right blue axis, blue dots and line) during the long-term $NOBF_4$ reaction.

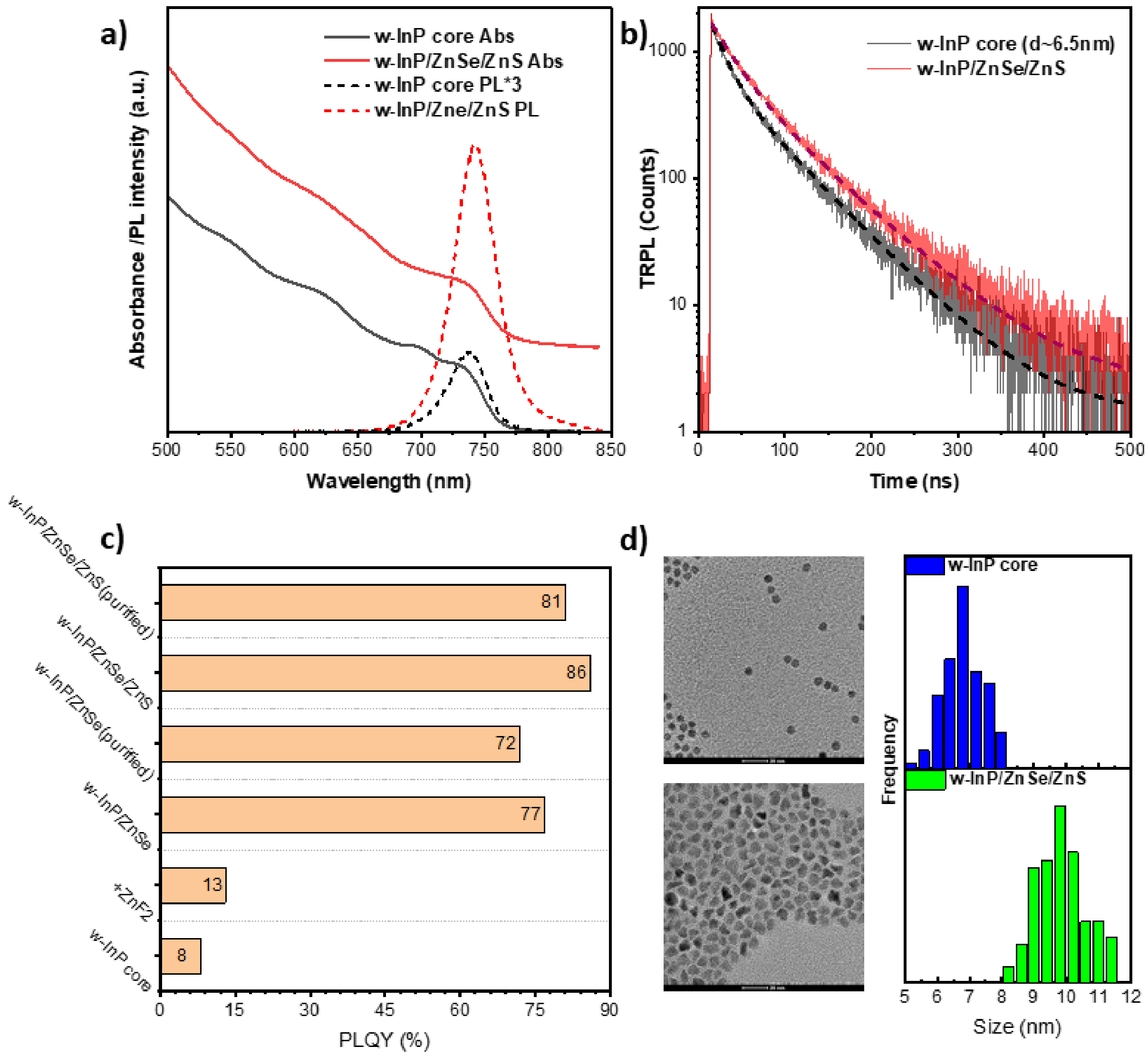

Figure S14. Preparation of of w-InP QDs with diameter of **6.8 nm** and surface passivation via ZnSe/ZnS shells: (a-c) optical properties: absorption, PL, TRPL spectra and PLQY measurements during synthesis of w-InP/ZnSe/ZnS QDs (d) size characterization: TEM images and corresponding diameter histograms, of 6.5 nm w-InP core (after $NOBF_4$ reaction) and resulting w-InP/ZnSe/ZnS QDs.

Table S4. Summary of parameters in terms of TRPL spectra for w-InP cores (d~6.8 nm) and resulting CSS QDs (using bi-exponential fitting model)

	PLQY (%)	τ_1 (ns)	A_1	τ_2 (ns)	A_2	τ_{avg} (ns)
w-InP core	8	17.2	0.598	62.2	0.402	49
w-InP/ZnSe/ZnS	81	26.8	0.528	69.3	0.472	56

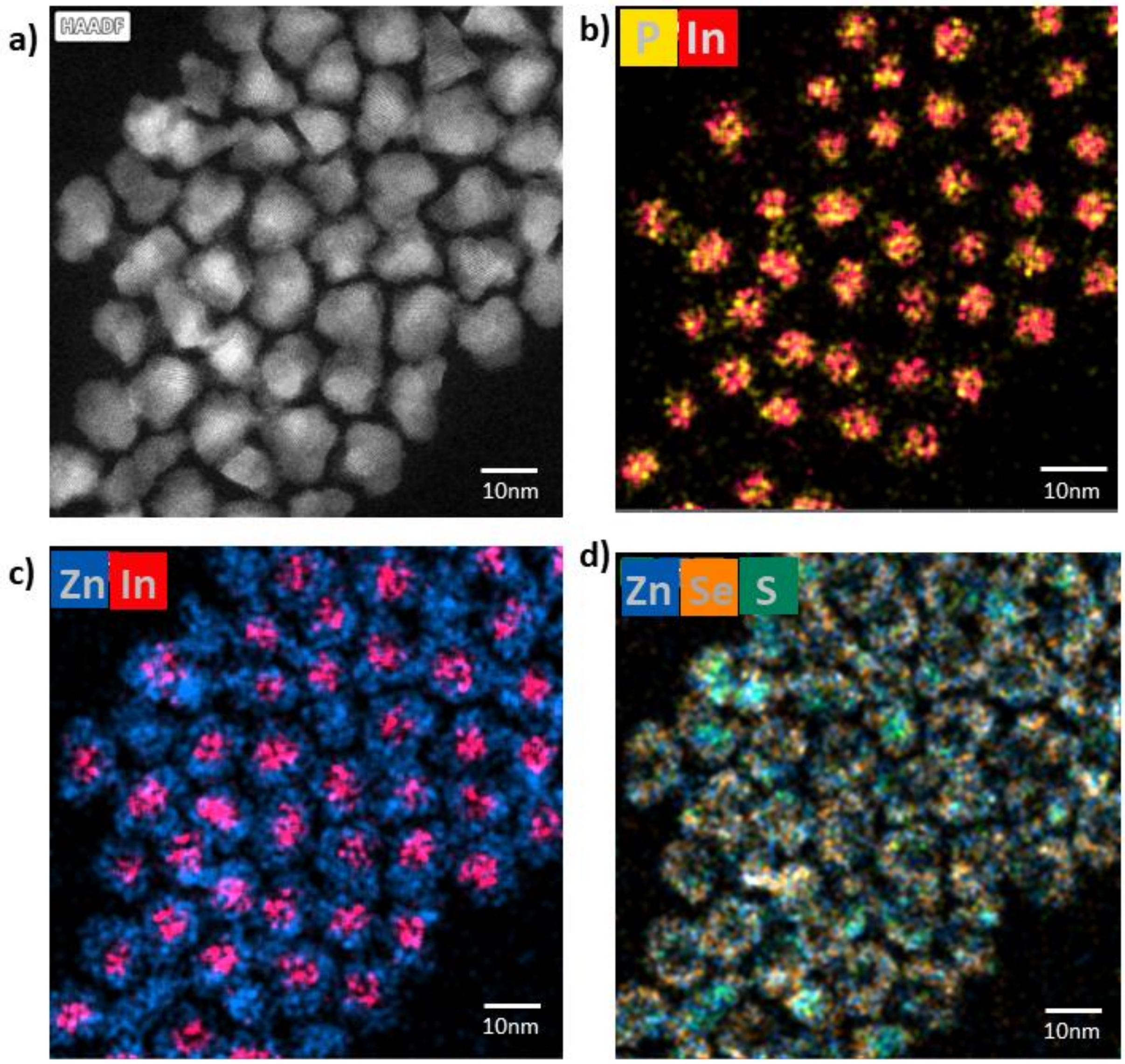

Figure S15. Structural characterization of w-InP/ZnSe/ZnS QDs based on the core diameter of **6.8 nm**: (a-d) HAADF-STEM image of w-InP/ZnSe/ZnS and corresponding mapping of elements of multiple QDs based on EDX scan. b In and P. c In and Zn. d Zn, Se and S.

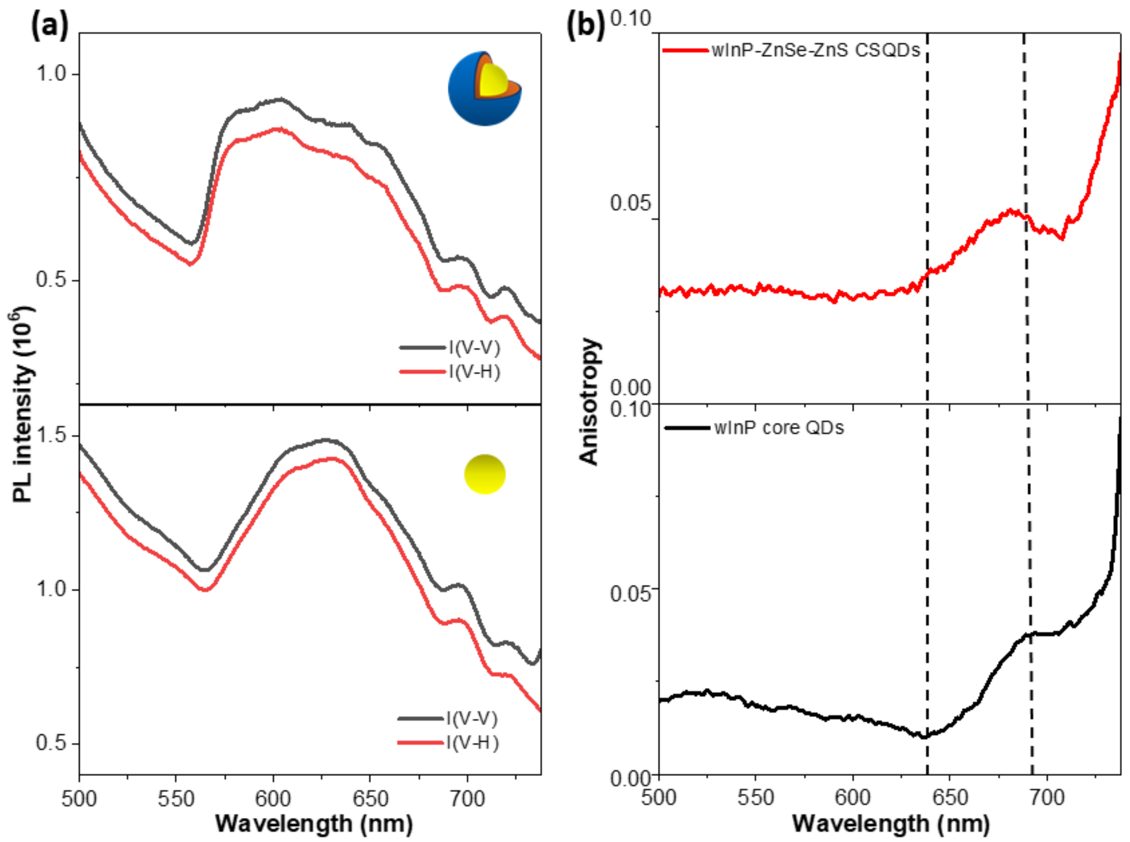

Figure S16. Luminescent anisotropic measurements of **6.8 nm** w-InP core (bottom) and corresponding w-InP/ZnSe/ZnS QDs (top) (bandgap emission of **~738nm** / 1.69eV): (a) PLE spectra of core and CSS QDs, with excitation and emission in parallel (VV) and in perpendicular (VH) arrangements. (b) Resulting PLE anisotropy spectra

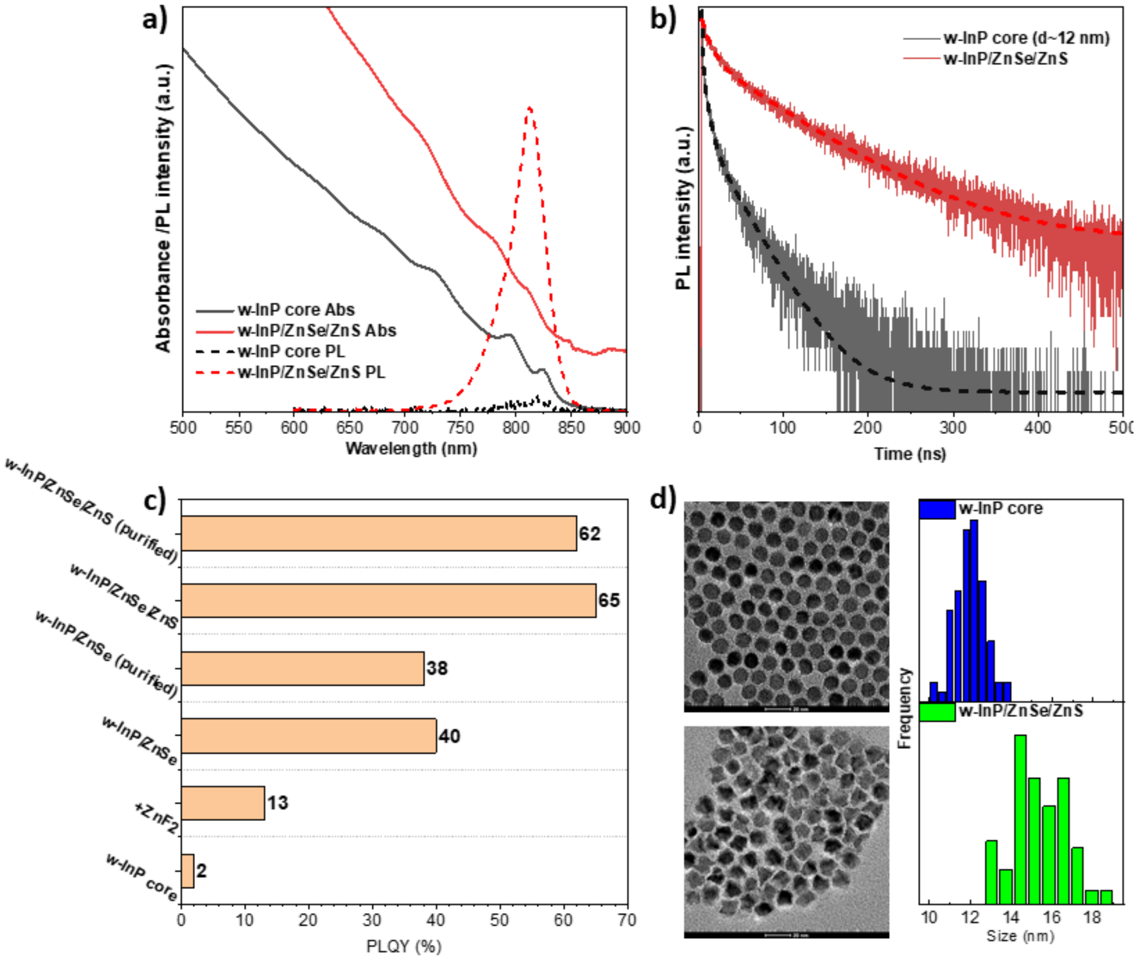

Figure S17. Preparation of of w-InP QDs with diameter of **12.0 nm** and surface passivation via ZnSe/ZnS shells: (a-c) optical properties: absorption, PL, TRPL spectra and PLQY measurements during synthesis of w-InP/ZnSe/ZnS QDs (d) size characterization: TEM images and corresponding diameter histograms, of 12.0 nm w-InP core (after $NOBF_4$ reaction) and resulting w-InP/ZnSe/ZnS QDs.

Table S5. Summary of parameters in terms of TRPL spectra for w-InP cores (d~12.0 nm) and resulting CSS QDs (using bi-exponential fitting model)

	PLQY (%)	τ_1 (ns)	A_1	τ_2 (ns)	A_2	τ_{avg} (ns)
w-InP core	2	5.48	0.796	41.8	0.204	29
w-InP/ZnSe/ZnS	62	18.6	0.519	120	0.481	105

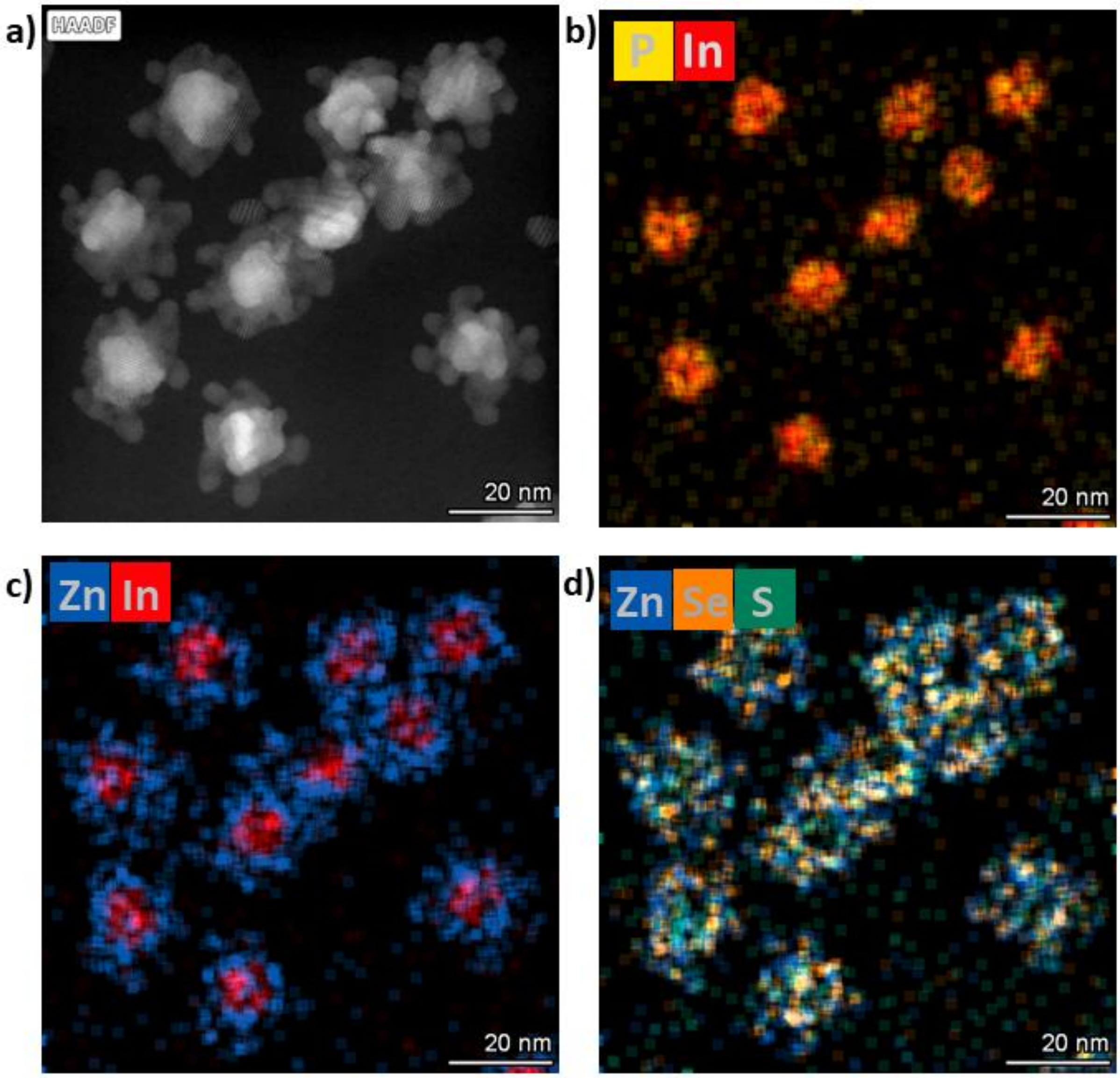

Figure S18. Structural characterization of w-InP/ZnSe/ZnS QDs based on the core diameter of **12.0 nm**: (a-d) HAADF-STEM image of w-InP/ZnSe/ZnS and corresponding mapping of elements of multiple QDs based on EDX scan. b In and P. c In and Zn. d Zn, Se and S.
